# Wearable full-body motion tracking of activities of daily living predicts disease trajectory in Duchenne muscular dystrophy

**DOI:** 10.1038/s41591-022-02045-1

**Published:** 2023-01-19

**Authors:** Valeria Ricotti, Balasundaram Kadirvelu, Victoria Selby, Richard Festenstein, Eugenio Mercuri, Thomas Voit, A. Aldo Faisal

**Affiliations:** 1 National Institute for Health and Care Research Great Ormond Street Hospital Biomedical Research Centre/University College London Great Ormond Street Institute of Child Health, London, UK; 2 Great Ormond Street Hospital for Children NHS Foundation Trust, London, UK; 3 Brain & Behaviour Lab, Department of Bioengineering, Imperial College London, London, UK; 4 Brain & Behaviour Lab, Department of Computing, Imperial College London, London, UK; 5 Behaviour Analytics Lab, Data Science Institute, Imperial College London, London, UK; 6 Gene Control Mechanisms & Disease Group Department of Brain Sciences, Imperial College London, London, UK; 7 Institute of Neurology, University College London, National Hospital for Neurology and Neurosurgery (University College London Hospitals), London, UK; 8 Medical Research Council London Institute of Medical Sciences, London, UK; 9 Università Cattolica del Sacro Cuore, Rome, Italy; 10 Policlinico Universitario Agostino Gemelli University Hospital, Rome, Italy; 11 Chair in Digital Health, Faculty of Life Sciences, University of Bayreuth, Bayreuth, Germany; 12 Brain & Behaviour Lab, Institute of Artificial & Human Intelligence, University of Bayreuth, Bayreuth, Germany

**Keywords:** Neuromuscular disease, Machine learning

## Abstract

Artificial intelligence has the potential to revolutionize healthcare, yet clinical trials in neurological diseases continue to rely on subjective, semiquantitative and motivation-dependent endpoints for drug development. To overcome this limitation, we collected a digital readout of whole-body movement behavior of patients with Duchenne muscular dystrophy (DMD) (*n* = 21) and age-matched controls (*n* = 17). Movement behavior was assessed while the participant engaged in everyday activities using a 17-sensor bodysuit during three clinical visits over the course of 12 months. We first defined new movement behavioral fingerprints capable of distinguishing DMD from controls. Then, we used machine learning algorithms that combined the behavioral fingerprints to make cross-sectional and longitudinal disease course predictions, which outperformed predictions derived from currently used clinical assessments. Finally, using Bayesian optimization, we constructed a behavioral biomarker, termed the KineDMD ethomic biomarker, which is derived from daily-life behavioral data and whose value progresses with age in an S-shaped sigmoid curve form. The biomarker developed in this study, derived from digital readouts of daily-life movement behavior, can predict disease progression in patients with muscular dystrophy and can potentially track the response to therapy.

## Abstract (web summary)

A digital biomarker derived from a multisensor bodysuit that monitors whole-body movement while individuals perform everyday activities can predict disease progression in Duchenne muscular dystrophy.

## Main

Advanced medicines including gene and cell therapies are rapidly emerging as disease-modifying treatment routes for rare or degenerative diseases. Drug development, however, is complicated mainly because clinical trials are hampered by the need for large cohorts difficult to realize in rare disease and because of the clinical bias and imprecision inherent to currently used trial endpoints. In many genetic and degenerative diseases, primary trial endpoints are still typically focused on behavioral assessments—‘by eye’ observations—of patients’ functional capability that predate the invention of computers. Yet, artificial intelligence (AI) is now a major driving force behind the rapid advances in digital healthcare^1^ by enabling more objective, data-driven approaches to understand^2^ and treat disease^3^. Digital biomarkers^4^, defined as objective, quantifiable data measured by means of digital devices, have recently seen increasing applications in clinical trials to overcome the intra- and interrater errors caused by subjective clinical scales. However, these digital biomarkers are not making full use of the true power of AI and data revolution in healthcare because they often just measure surrogates of existing markers, such as the number of steps or distance walked, markers originally chosen by human observer bias. Thus, they merely digitally replicate the ability of human observers, instead of embracing the possibility of going above and beyond human perceptual capabilities by looking at vastly more data in more detail. Human movement behavior is highly variable and complex^5^ and so the data are difficult to analyze, which is why traditional clinical trial endpoints using conventional or even new digital methods have focused for decades on old human-defined outcome measures.

We focused on the combination of wearable sensor technology and machine learning methods to overcome these limitations. We hypothesized that these methods would allow us to identify barely perceptible complex patterns in patient movement behavior, thus overcoming clinical or observer bias^6^. To this end, we also needed to overcome a limitation of observational endpoints, namely that they require preprescribed activities that have been historically believed to be useful, such as ‘walking for 6 min’ or ‘touching the tip of the nose’, but have never been grounded in how relevant these activities are for patients in their daily life. Thus, we applied our ability to define with AI a digital biomarker by not only relying on prescribed observational assessment activities but also by challenging ourselves to use information from unconstrained datasets, by simply observing patients performing activities of daily living (ADLs). In the same way that systematic approaches in comprehensively collecting a full picture of the genome sparked the genomics revolution, we believe that systematic approaches in ‘sequencing’ and understanding human natural movement behavior (that is, human ethology in health and disease) could allow us to pursue an approach^7–9^. Therefore, we pursue here the conception of ethomic biomarkers for human clinical use. We hypothesized that AI could fundamentally change disease characterization, develop objectively quantifiable readouts and thereby reduce necessary cohort sample size and time- to-endpoint. We used DMD, the most common fatal neuromuscular disease with a mean survival into the late second to third decade^10–13^ as a model because over 80 clinical trials are currently active in DMD^14^ and a number of these are microdystrophin gene transfer clinical trials^15–17^. Drug development for DMD has proven a particularly stony path due to the relentless degeneration of the skeletal and heart muscles but also due to methodological standstill of trial methodology largely using disease state-specific, observer- and motivation-dependent endpoints^18–22^.

## Results

### Overview of the approach

**Fig. 1:**
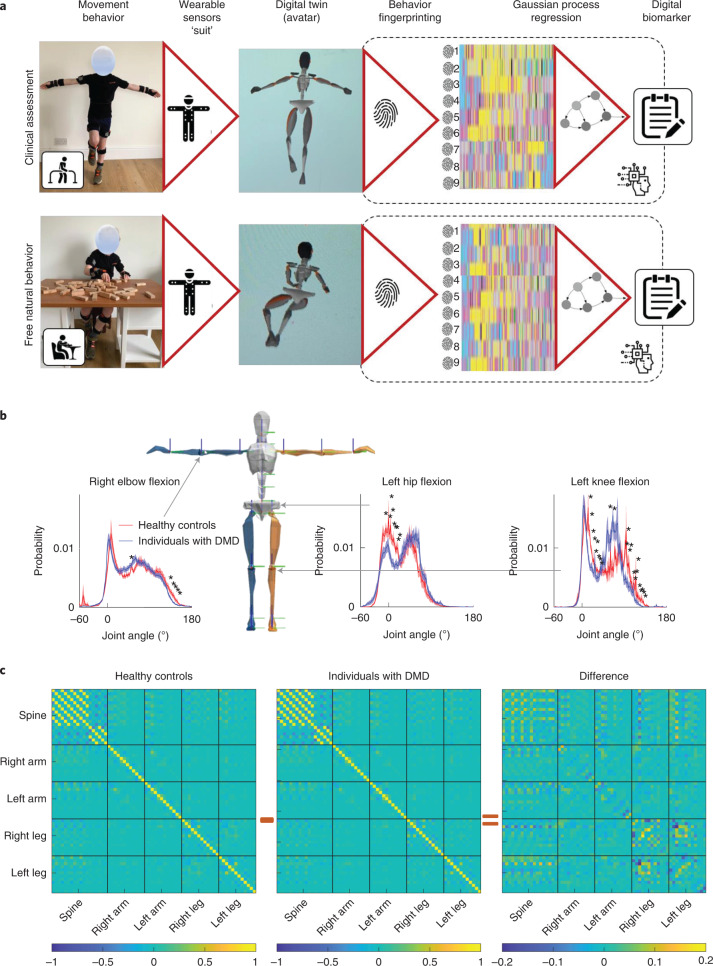
Overview of ethomic fingerprinting of natural movement behavior of individuals with DMD and healthy controls. **a**, Overview of our system, in which ethomic fingerprints were extracted from the digital twin (avatar) of a participant, which was created using the suit data of natural movement behavior. A supervised machine learning algorithm, GP regression, was then used to derive the digital biomarker from the ethomic fingerprints. **b**, Probability distribution of joint angles at three exemplary skeletal joints (from left to right: right elbow flexion, left hip joint flexion, left knee joint flexion) for natural movement behavior data of individuals with DMD (blue) and healthy controls (red). Shaded regions denote the standard error and the stars denote a significant difference (*P* < 0.05, Kruskal–Wallis one-way ANOVA). **c**, Holistic view of whole-body coordination in ADLs. The correlation matrix (that is, the Pearson correlation coefficient of the joint angular velocities) for major skeletal joints is shown for healthy controls (left) and individuals with DMD (middle); the difference is shown on the right. The value in the color bars corresponds to the Pearson correlation coefficient. For a complete list of the joints for which movement data were obtained, see Supplementary Table 3.

In our approach (Fig. 1a) we developed a fully data-driven, whole-body movement behavior analytics methodology to derive new digital biomarkers. We strove to develop an approach that is objective, quantifiable, investigator- and motivation-independent and that can seamlessly capture motor performance from early childhood to adulthood, thereby reliably recording the whole-body kinematic behavior of an individual in spontaneous movement across the entire disease trajectory.

Our clinical trial included two cohorts (see Methods for the full trial details): male individuals with DMD and age/sex-matched healthy controls (HC). Clinical diagnosis of DMD was genetically confirmed by multiplex ligation-dependent probe amplification (MLPA), full gene sequencing or any other state-of-the-art diagnostic technique. At recruitment, 21 males with DMD with a mean age 9.4 years (range: 6–17 years), 18 ambulant and 3 non-ambulant were included in this study. Furthermore, 17 age-matched healthy controls, mean age 10.4 years (range: 4–16 years) were recruited. Individuals with DMD and healthy controls were followed over the course of 12 months and were examined at baseline, 6 and 12 months at Great Ormond Street Hospital (healthy controls at baseline and 12 months). Each visit involved the performance of the three clinical assessments: 6-min walk distance test (6MWD), North Star Ambulatory Assessment (NSAA) and the Performance of the Upper Limb (PUL) test, which are all used as primary endpoints in most clinical trials of DMD (Methods) as well as capturing the patients’ unconstrained daily-life movement behavior before and after their assessments on that day. Our cohort with DMD did not undergo any magnetic resonance imaging (MRI) scans on the day of the assessment.

We have used wearables to systematically quantify movement behavior in its complexity by using full-body motion capture allowing full freedom of range and movement (that is, behaviorally ‘sequencing’ the normal movement behavior of individuals). Our approach allowed us to select distinguishing behavioral fingerprints in a small cohort of patients with DMD (*n* = 21). We compared these fingerprints against age-matched healthy controls (*n* = 17), established their cross-sectional and longitudinal predictive capacity using machine learning and tested the effectiveness of our predictions against the current standard observational approach using data from a separate, larger natural history study in children with DMD (*n* = 44), referred to as the Gemelli study.

### Ethomic fingerprints

**Extended Data Fig. 1:**
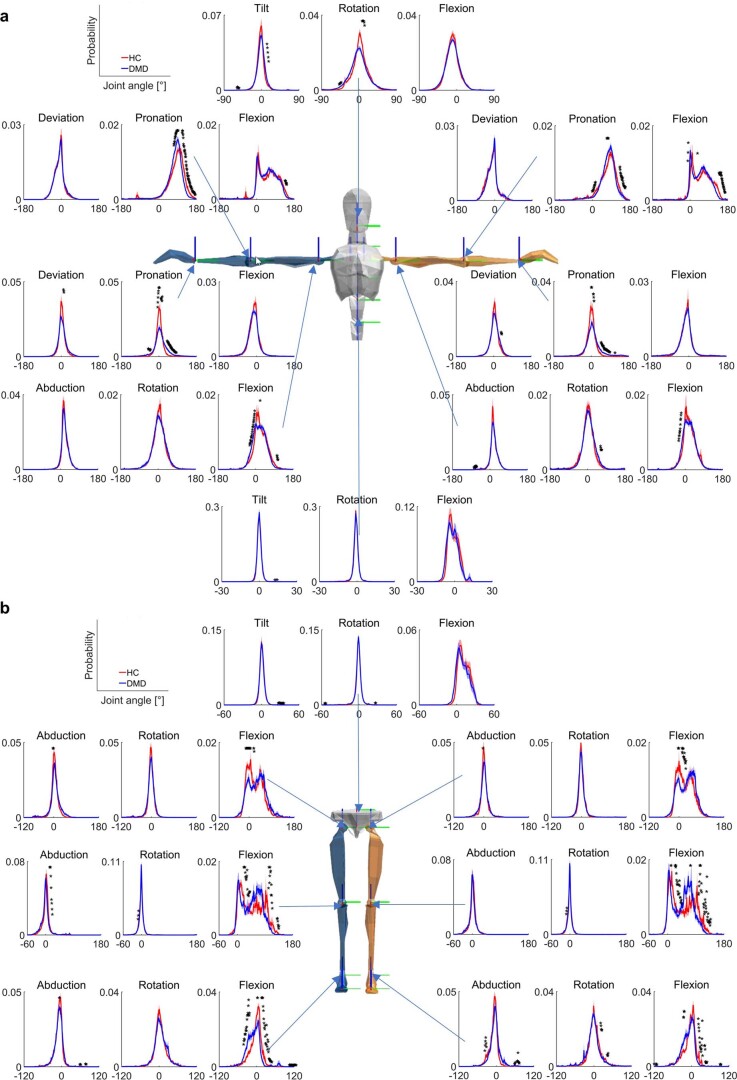
Probability distribution of angular displacements. **a**. and **b**. Probability distribution of angular displacements of the joints of the upper body and the lower body for natural movement behaviour data of DMD subjects (21 subjects and 46 visits) and healthy controls (17 subjects and 21 visits).

In the following, we describe the development and validation of our approach step by step. Since we did not want to impose assumptions about what elements of movement of the body were more important, we simply collected movement data from the whole body and later used the data to find what was best suited for characterizing the disease, thereby avoiding observer bias. We used a wearable sensor ‘suit’ (a set of wristwatch-sized (4.7 × 3.0 × 1.3 cm) sensors attached with Velcro to the body or clothing of the individuals) that allowed us to capture the motion trajectory of all limbs and the body—from foot to hip, from hand to shoulder and from hip to head at a temporal resolution of 60 Hz. In the first step, we compared the joint kinematics of ADLs between healthy controls and patients with DMD and already found significant distinguishing elements: the distribution of joint angles across almost all body joints showed differences between DMD and controls (Fig. 1b and Extended Data Fig. 1). These quantitative differences are consistent with the qualitative descriptions of DMD in several ways. First, by inspecting the histograms of skeletal joint movement throughout ADLs (Fig. 1b and Extended Data Fig. 1 for all major joints) we saw reflection of hyperlordosis in the DMD posture: the right shifted distribution of the angles subtended by the hip joint with respect to healthy controls and correspondingly a stronger flexion (left shift of the distribution) of the knee joint. Similarly, the overall stiffer posture in DMD upper-body poses is reflected by the more contracted distribution of the DMD elbow joint angles compared to healthy controls. Second, the characteristic DMD Trendelenburg sign (waddling gait) is reflected in the joint angular velocity correlation matrix of ADLs (Fig. 1c) where across the lower extremities we saw less anticorrelation between joints moving in the sagittal plane (knee and hip flexion) and more correlation in the coronal plane (sideways abduction of the hips) that reflect waddling.

**Fig. 2:**
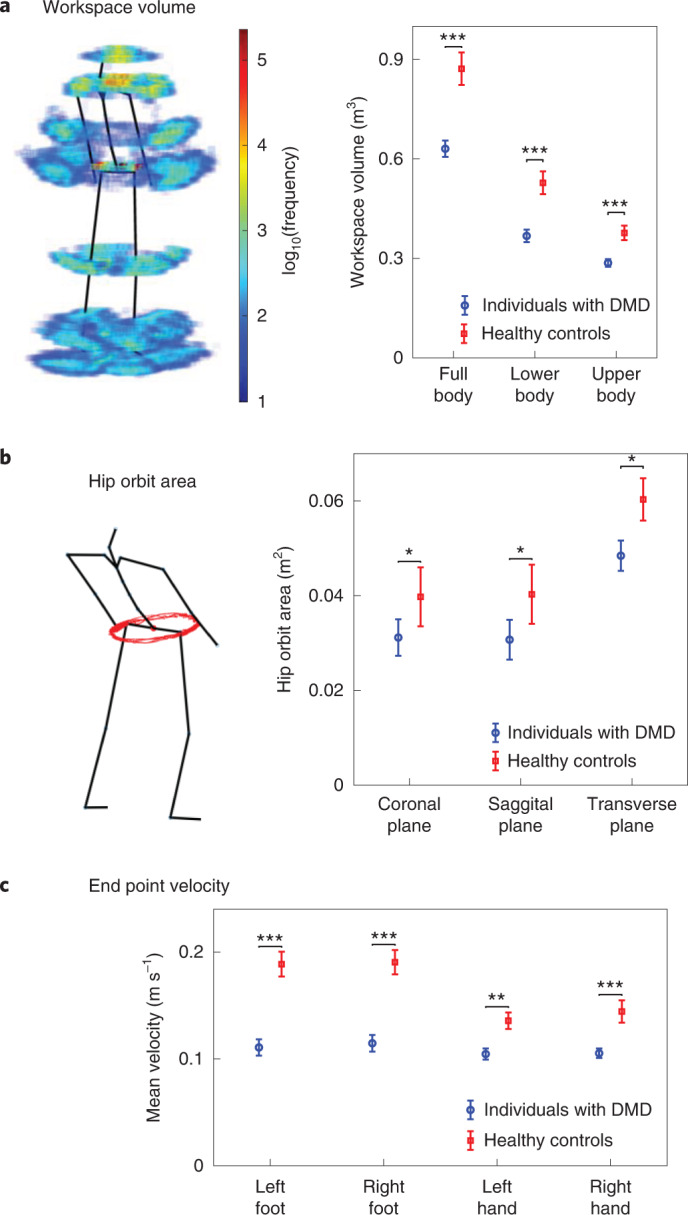
Three exemplary ethomic fingerprints. **a**, Left: workspace volume density plot generated using the 3D location of joints from a typical individual. Right: comparison of the workspace volume of individuals with DMD and healthy controls. The space is segmented in blocks of size 2 × 2 × 2 cm and the color of each block represents the frequency (shown on a log_10_ scale in the color bar) of any joint visiting the block. The workspace volume of the healthy controls was significantly greater than that of individuals with DMD (Kruskal–Wallis one-way ANOVA, where *P ≤ 0.05, **P ≤ 0.01 and ***P ≤ 0.001). **b**, Left: hip trajectory of a typical individual with DMD. Right: comparison of the hip orbit area of individuals with DMD and healthy controls. The hip orbit area of ambulatory individuals with DMD was significantly lower compared to the healthy controls (*P* < 0.05, Kruskal–Wallis one-way ANOVA). **c**, Comparison between the mean velocities of the extremities of individuals with DMD and healthy controls. The mean velocity of healthy controls was significantly greater than that of individuals with DMD for all four extremities (*P* < 0.01, Kruskal–Wallis one-way ANOVA). **a**–**c**, Data are presented as the mean ± standard error. (*n* = 46 visits of individuals with DMD (41 ambulatory) and *n* = 21 healthy control visits). For a complete list of movement behavioral fingerprinting features and the exact *P* values of the Kruskal–Wallis one-way ANOVA tests, see Supplementary Table 5.

**Extended Data Fig. 2:**
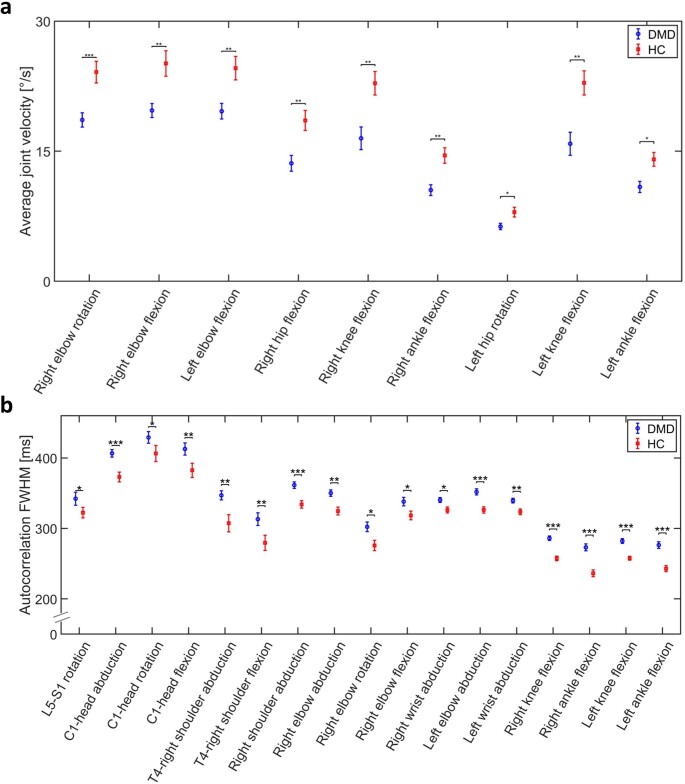
Ethomic fingerprints – Average joint velocity and autocorrelation full width at half-maximum. **a**. The DMD subjects exhibit significantly slower average velocities in all joints when compared to healthy controls. **b**. The autocorrelation full width at half-maximum (FWHM) of the body joints shows a significantly slower drop for the healthy controls than DMD subjects indicating that the movements of the DMD subjects are changing more slowly compared to the healthy controls. Kruskal-Wallis one-way ANOVA, where ∗ represents p ≤ 0.05, ∗∗ is p ≤ 0.01 and ∗∗∗ is p ≤ 0.001. In **a**-**b**, data are presented as mean ± standard error (n = 46 DMD visits and n = 21 HC visits). For the exact p-values of the Kruskal-Wallis one-way ANOVA tests, see Supplementary Table 5.

**Extended Data Fig. 3:**
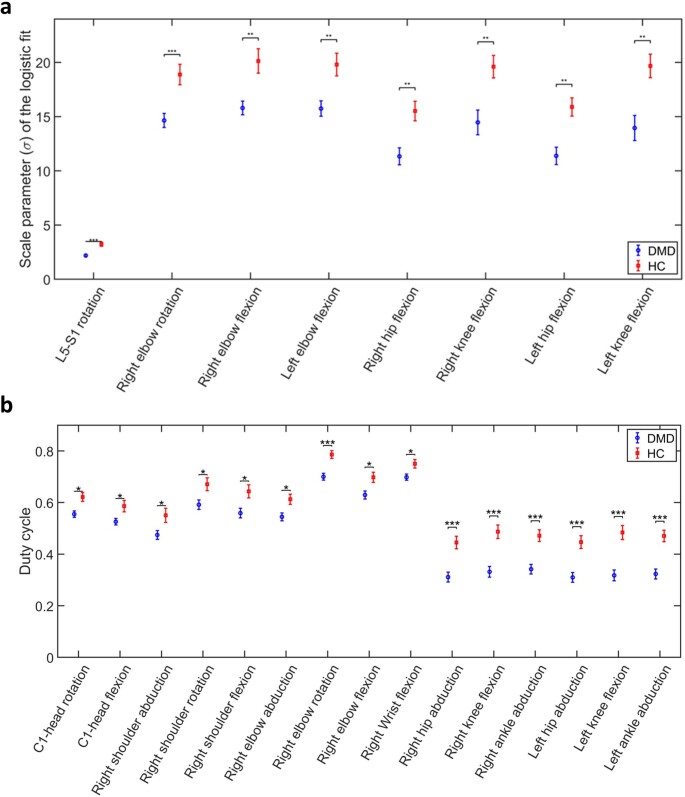
Ethomic fingerprints – Scale parameter of the logistic fit and duty cycle of joint velocities. **a**. A comparison between the scale parameter (σ) of the logistic fit of the joint velocities between the DMD subjects and healthy controls show a significantly larger σ in the joints of the healthy controls meaning that they used a much wider range of velocities than the DMD subjects **b**. A comparison between the duty cycle of the joints of the DMD subjects and healthy controls shows that the duty cycle of the healthy controls is significantly greater than that of the DMD subjects for many joints indicating that healthy controls move their joints more often than the DMD subjects. Kruskal-Wallis one-way ANOVA, where ∗ represents p ≤ 0.05, ∗∗ is p ≤ 0.01 and ∗∗∗ is p ≤ 0.001. In **a**-**b**, data are presented as mean ± standard error (n = 46 DMD visits and n = 21 HC visits). For the exact p-values of the Kruskal-Wallis one-way ANOVA tests, see Supplementary Table 5.

**Extended Data Fig. 4:**
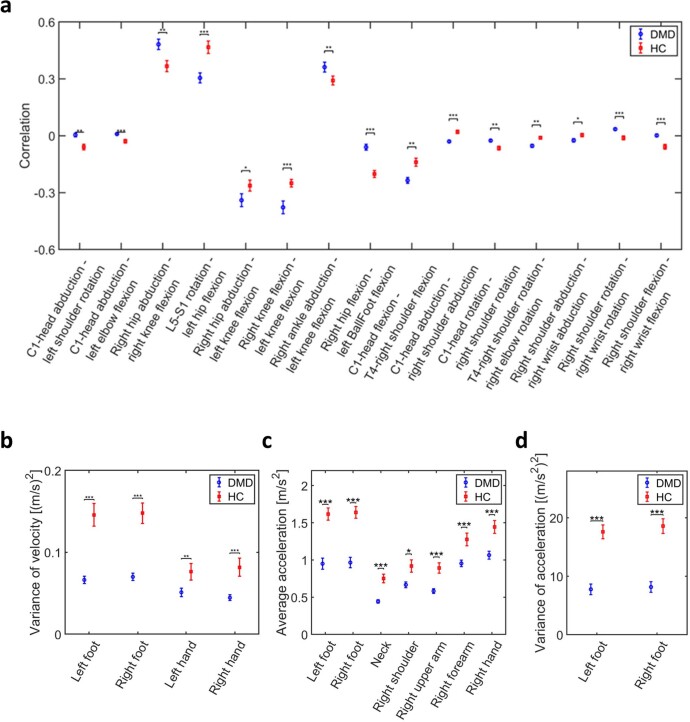
Ethomic fingerprints – Correlation of joint velocities and body segment velocities and acceleration. **a**. A comparison between the Pearson’s correlation coefficient of the angular velocities of the joints between the DMD subjects and healthy controls show significant differences. **b**. A comparison between the variance of velocity of the extremities of the DMD subjects and healthy controls shows that the variance of the velocity of the healthy controls is significantly greater than that of the DMD subjects for all four extremities **c**. and **d**. A comparison between the mean (**c**) and variance (**d**) of the acceleration of the different body segments of the DMD subjects and healthy controls shows that the both mean and variance of the acceleration of the healthy controls is significantly greater than that of the DMD subjects Kruskal-Wallis one-way ANOVA, where ∗ represents p ≤ 0.05, ∗∗ is p ≤ 0.01 and ∗∗∗ is p ≤ 0.001. In **a**-**d**, data are presented as mean ± standard error (n = 46 DMD visits and n = 21 HC visits). For the exact p-values of the Kruskal-Wallis one-way ANOVA tests, see Supplementary Table 5.

**Extended Data Fig. 5:**
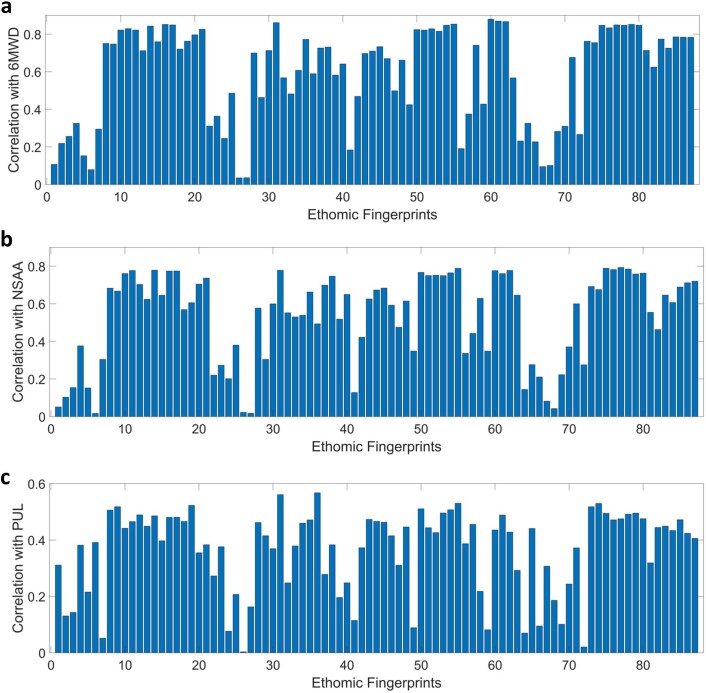
Correlation between the ethomic fingerprints and the standard clinical scales. Bar chart of the Pearson correlation between each feature in the full set of ethomic fingerprints listed in Supplementary Table 5 and the clinical scales of 6 MWD (**a**), NSAA (**b**) and PUL (**c**).

We then moved from descriptive statistics to more principled data-derived fingerprints of human movement behavior (Methods). These fingerprints characterized movement trajectories of the full body (that is, all joint angles). Specifically, our fingerprints were not meant to measure any specific activity (such as walking) but characterize human movement behavior in a more holistic way. Fingerprints included the mean velocities of the extremities, the hip movement orbit and the volumetric workspaces of various joints (Fig. 2a–c for examples and Table 1 and Methods for a list of all identified fingerprints). Importantly, some of our movement behavior fingerprints have not been described in the context of DMD before (see Ethomic fingerprints subsection of Methods). Digital fingerprinting of movement behavior involves applying each fingerprint to the time series of movements and obtaining the strength of its presence. Each fingerprint could individually already distinguish individuals with DMD from healthy controls and correlated well with clinical scales (Extended Data Figs. 2–5 for their results).

**Table 1:** Overview of the ethomic fingerprints

No.	Name of ethomic fingerprint	Description	No. of dimensions in each fingerprint
1	Workspace volume	Volume occupied by the joints calculated using the 3D location of the joints	3
2	Hip orbit area	Area generated by the hip movement in the 3 planes	3
3	Extremities velocity	Average and variance of the velocities of the extremities (hands and feet) in space	8
4	Average joint velocity	Average of the joint angular velocities	9
5	Autocorrelation full-width at half-maximum	Measure of the rate of change in individual joint angles	17
6	Variability of the joint angle velocities	Scale parameter of the logistic distribution of the joint angular velocities	8
7	Skeletal joints linear correlations	Pearson correlation coefficient of the joint angular velocities	15
8	Duty cycle	Fraction of time a joint is in motion	15
9	Acceleration of the body segments	Average and variance of the acceleration of the body segments	9

Please see the Methods for the definitions of the fingerprints, Supplementary Table 5 for their detailed list and Fig. 2 and Extended Data Figs. 2–4 for the results for each fingerprint.

### Cross-sectional predictions of clinical scales

**Fig. 3:**
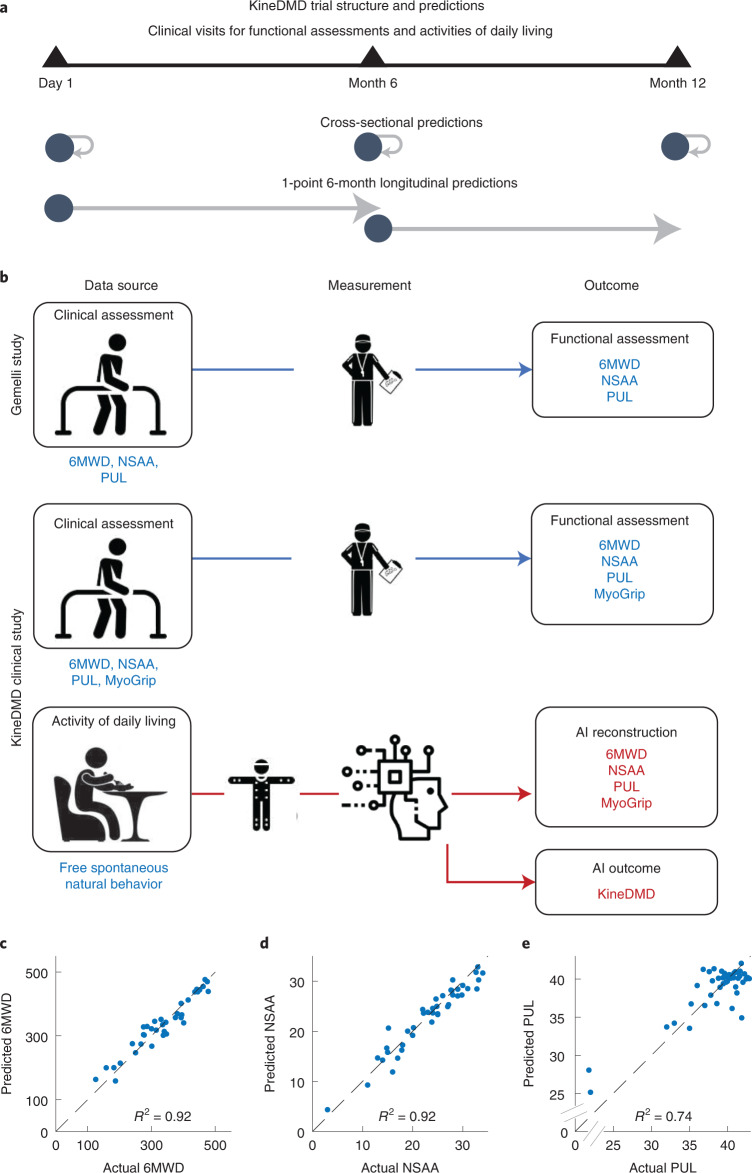
KineDMD trial structure and cross-sectional predictions. **a**, Pictorial representation of the KineDMD clinical trial structure and the different types of predictions done in the study. **b**, Pictorial representation of the Gemelli and KineDMD studies showing the data source, measurement method and outcomes. **c**–**e**, Plots of actual versus predicted values for 6MWD (**c**), NSAA (**d**) and PUL (**e**). Cross-sectional predictions of these clinical scales were made using the ethomic fingerprints. Each point represents the actual versus predicted score for a patient’s visit. A LOSO cross-validation and Gaussian process regression were used to find a mapping between the ethomic fingerprints from the natural movement behavior of the patients with DMD and the different clinical scales of 6MWD (*n* = 17 ambulatory individuals, 37 visits), NSAA (*n* = 18 ambulatory individuals, 41 visits) and PUL (*n* = 21 individuals, 45 visits).

**Extended Data Fig. 6:**
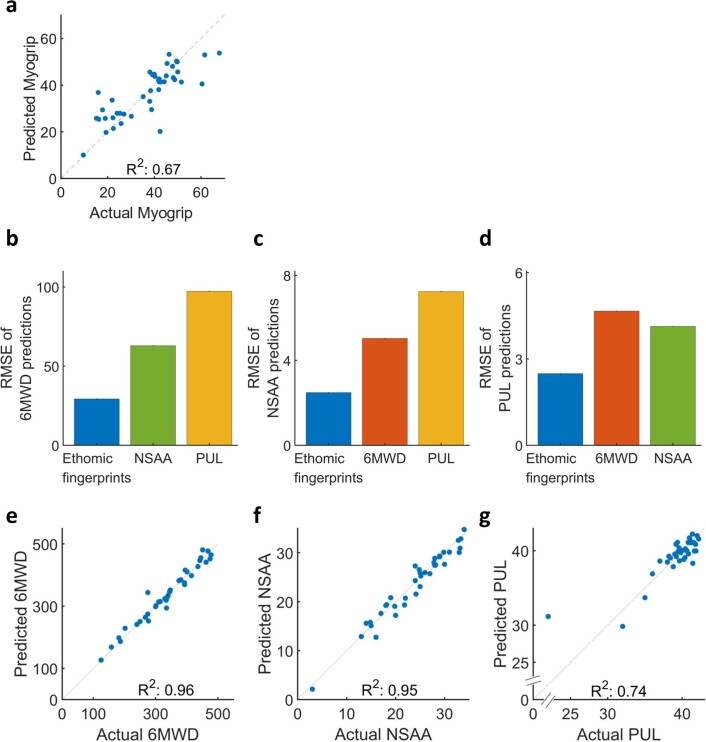
Cross sectional predictions of the clinical scales. **a**. Cross sectional prediction of Myogrip. Actual vs predicted plot of the Myogrip. Each point represents the actual vs predicted score for a patient’s visit. A leave-one-subject-out cross-validation (n = 21 subjects, 44 visits) and Gaussian process regression was used to find a mapping between the ethomic fingerprints from the natural movement behaviour of the DMD patients and the Myogrip. **b-d**. Cross-sectional predictions of the clinical scales. Comparison of the aggregate RMSE of the leave-one-subject-out cross-validated predictions (n = 17 ambulatory subjects, 37 visits) of the 6 MWD (**b**)using ethomic fingerprints vs other clinical scales. **c** and **d** are the corresponding plots for NSAA (n = 18 ambulatory subjects, 41 visits) and PUL (n = 21 subjects, 45 visits) respectively. **e-g**. Cross-sectional predictions using 6 MW data. Actual vs predicted plot from the cross-sectional prediction of the clinical scales using 6MW data. Each point represents the actual vs predicted score for a patient’s visit. A leave-one-subject-out cross-validation and Gaussian Process regression was used to find a mapping between the ethomic fingerprints from the 6MW motor behaviour of the DMD patients and the different clinical scales of 6MWD, NSAA and PUL (n = 17 ambulatory subjects, 37 visits).

We then asked if we could make cross-sectional predictions from real-life-derived movement behavioral fingerprints to predict clinical scales (Fig. 3a top for cross-sectional test points in our trial), and if confirmed, extrapolate to longitudinal predictions (Fig. 3a bottom for how we mapped the longitudinal test points to our trial). Crucially, our ethomic fingerprints can be applied to any human kinematic activity. This means we can use it to characterize structured functional assessments but also characterize unstructured-unconstrained ADLs giving us a measure in a common ‘currency’ of motor capability across all these possible activity settings (Fig. 3b). Indeed, each of our ethomic fingerprints showed individually no or only little overlap between healthy controls and individuals with DMD (Extended Data Figs. 2–4). This could be further improved by combining all the individual ethomic fingerprints via state-of-the-art supervised machine learning using Gaussian process regression (Methods, Gaussian process regression). This allowed us to use unstructured movement behavior data of ADLs of children to predict at a cross-sectional level three very different clinical functional assessments (Fig. 3c): 6MWD (*R*^2^ = 0.92, root-mean-squared-error (RMSE) = 26.74); NSAA (*R*^2^ = 0.92, RMSE = 1.98); and PUL (*R*^2^ = 0.74, RMSE = 2.20). We have also included our successful results (*R*^2^ = 0.67, RMSE = 7.82) for a new force-based biomarker, MyoGrip (Extended Data Fig. 6a). Further, we were also successful at predicting the clinical scales from the ethomic fingerprints from the 6MW test data alone (Extended Data Fig. 6c for the results).

We tested if any of the three criterion standard clinical functional assessments could be used to predict each other’s scores (Extended Data Fig. 6b) but the criterion standard measures were not good at predicting each other’s scores; only our ethomic fingerprints could predict each and all three. (See Supplementary Figs. 1–3 for the fingerprints selected by the feature selection algorithm for cross-sectional predictions of the clinical scales.) This confirmed that our digital fingerprinting could at least recreate criterion standard measures in terms of performance, making it a useful tool to provide digital versions of the clinical functional assessments but also suggesting that it captured more information about disease state than each and all criterion standard measures.

### Longitudinal predictions of clinical scales

**Fig. 4:**
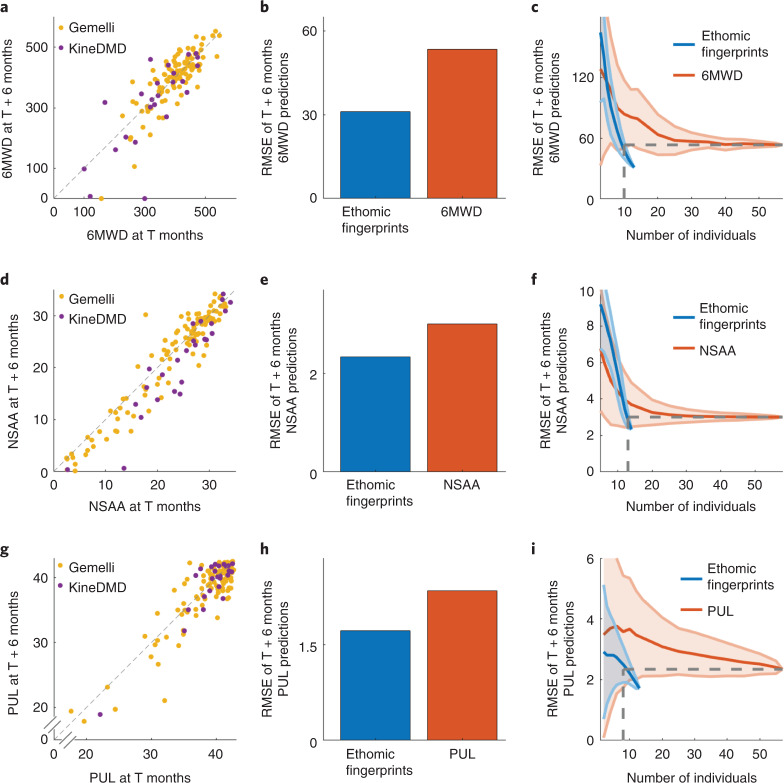
Longitudinal predictions of the clinical scales. **a**, Scatter plot of the 6MWD from the visit at time T against the 6MWD from the visit at time T + 6 months from both the KineDMD study (*n* = 13 individuals with 24 longitudinal visits) and the Gemelli study (*n* = 44 individuals with 122 longitudinal visits). A small jitter was added to the points to show any overlapping points. **b**, Comparison of the aggregate RMSE of the LOSO cross-validated predictions of the 6MWD at T + 6 months by the ethomic fingerprints from visit T (blue bar) and the 6MWD at visit T (red bar). The LOSO cross-validated prediction of the 6MWD at T + 6 months using the 6MWD at T months was performed on the combined cohort of KineDMD (*n* = 13 individuals, 24 visits) and Gemelli studies (*n* = 44 individuals, 122 visits). The LOSO cross-validated prediction of the 6MWD at T + 6 months using the ethomic fingerprints at T months was performed on the KineDMD cohort (*n* = 13 individuals, 24 visits). **c**, Plot of the aggregate LOSO cross-validated RMSE of the predictions of the 6MWD at T + 6 months as a function of the number of individuals used to build the machine learning model. The model using ethomic fingerprints achieves better performance with a smaller number of individuals (marked by the vertical dotted lines) compared to the model using 6MWD from the combined data of the KineDMD and Gemelli studies. The shaded regions indicate the s.d. of the results from different models built for each number of individuals. **d**–**f**, Corresponding results for NSAA. **g**–**i**, Corresponding results for PUL.

**Extended Data Fig. 7:**
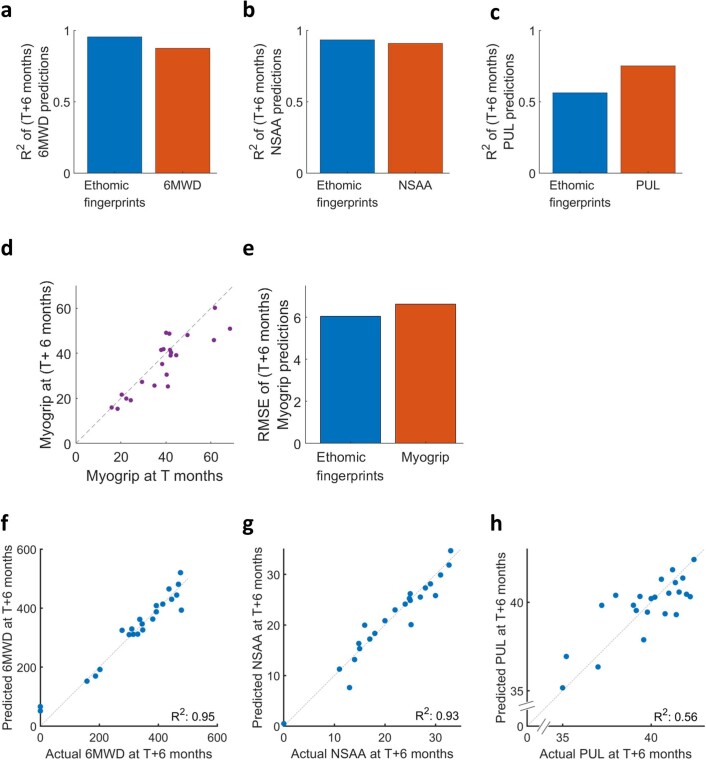
Longitudinal predictions of the clinical scales. **a**. Comparison of the aggregate R^2^ of the leave-one-subject-out cross-validated predictions of the 6MWD at T + 6 months by the ethomic fingerprints from visit T vs the predictions by 6MWD at visit T. The leave-one-subject-out cross-validated prediction of the 6MWD at T + 6 months using the 6MWD at T months (red bar) was done on the combined cohort of KineDMD study (n = 13 subjects with 24 longitudinal visits) and Gemelli study (n = 44 subjects with 122 longitudinal visits). The leave-one-subject-out cross-validated prediction of the 6MWD at T + 6 months using the ethomic fingerprints at T months (blue bar) was done on the KineDMD cohort (n = 13 subjects, 24 visits). **b** and **c** are the corresponding plots for NSAA and PUL respectively. **d**. Scatter plot of the Myogrip from the visit at time T against the Myogrip from a visit at time (T + 6 months) from KineDMD study (n = 13 subjects, 23 visits). Myogrip was not collected from Gemelli study. **e**. Comparison of the aggregate RMSE of the leave-one-subject-out cross-validated predictions of the Myogrip at T + 6 months by the ethomic fingerprints from visit T and Myogrip at visit T. **f-h**. Actual vs prediction plot from the longitudinal prediction models of the clinical scales 6MWD (**f**), NSAA (**g**) and PUL (**h**) using ethomic fingerprints. Each point represents the actual vs predicted score for a patient’s visit.

Next, we tackled the challenge of predicting longitudinal disease progression accurately, which is a key requirement of drug development (Fig. 4 and Extended Data Fig. 7d for MyoGrip). To build the longitudinal disease prediction models using the clinical scales of 6MWD, NSAA and PUL scores as predictors, we used the data from the combined cohort of the KineDMD study (*n* = 13 individuals, 24 visits) and the larger Gemelli study (*n* = 44 individuals, 122 visits) to allow a larger data size for the models using the clinical scales. First, we characterized the considerable amount of variability that patients show over 6 months in the criterion standard clinical scales in terms of disease progression (Fig. 4a,d,g). To compute the ability of criterion standard clinical scales (6MWD, NSAA and PUL) to carry information about disease evolution, we used Gaussian process regression to predict their scores 6 months into the future (Fig. 4b,e,h, red bar; 6MWD RMSE = 53.40; NSAA RMSE = 3.01; PUL RMSE = 2.34) with the clinical scales as predictors. This was in contrast to the ethomic fingerprinting-based predictions (Fig. 4b,e,h, blue bar; 6MWD RMSE = 31.08; NSAA RMSE = 2.34; PUL RMSE = 1.72), which were systematically more accurate in predicting the disease course over the next 6 months (Extended Data Fig. 7a–c for the *R*^2^ of the predictions). This implies that our approach of movement behavioral fingerprinting to use high-resolution kinematics from daily life contained sufficiently rich information not only to score the disease state of the patient in the present but also predict how the patient would evolve (Supplementary Figs. 4 and 5 for the fingerprints selected by feature selection algorithm for longitudinal predictions of the clinical scales).

We then plotted the error of the longitudinal predictions as a function of the number of individuals used to build the machine learning models (Fig. 4c,f,i). The models built using the ethomic fingerprints from a smaller number of individuals (*n* = 13 individuals, 24 visits) achieved a lower error compared to the models built using the clinical scales from a larger cohort from the combined KineDMD and Gemelli studies (*n* = 44 individuals, 122 visits). In addition, the prediction error obtained using the ethomic fingerprints was substantially smaller in variance (for number of individuals = 10, an s.d. of 8.13 versus 33.63 for 6MWD, 1.24 versus 1.87 for NSAA, 0.35 versus 1.67 for PUL) when compared to the prediction error obtained from the criterion standard clinical measures. Thus, both accuracy and precision are multiples higher for our ethomic fingerprints even when using a much smaller cohort, than those of the criterion standard clinical scales. By accurately predicting the disease progression of each individual patient, in the context of clinical trials (Extended Data Fig. 7f), the ethomic fingerprints may thus be used to quantify the degree of deviation from this trajectory in the context of a disease-modifying therapy. Moreover, our work establishes that a small population size is sufficient to build prediction models with high accuracy when using the rich set of ethomic fingerprints applied to natural data, which would considerably reduce the number of patients required in the context of drug development (Fig. 4c,f,i).

### AI-derived biomarker

**Fig. 5:**
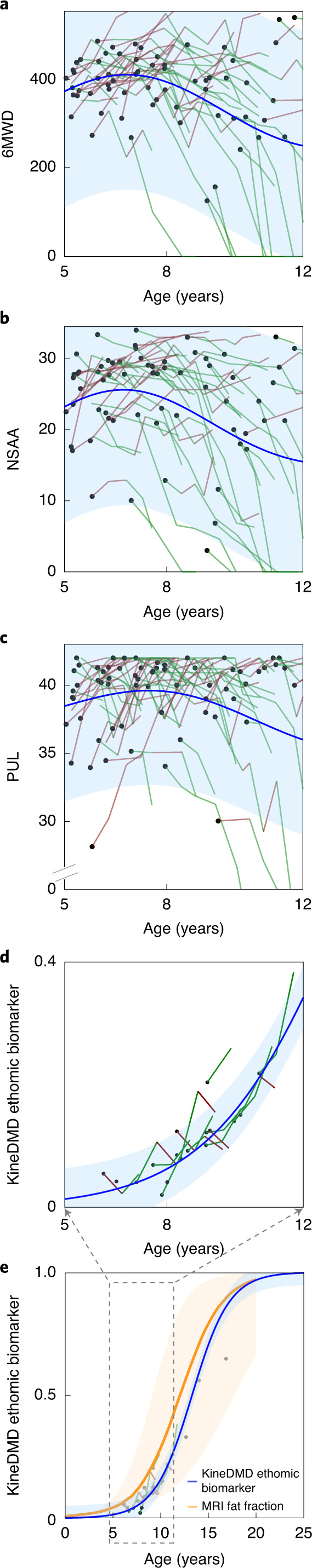
Current clinical scales and the KineDMD ethomic biomarker. **a**–**c**, Longitudinal history of the 6MWD (**a**), NSAA (**b**) and PUL (**c**) scores of individuals with DMD from the KineDMD and Gemelli studies as a function of age plotted on the *x* axis. The score at the first visit of the individual (black dots) and subsequent visits (lines flowing from the dot) shows the evolution of the clinical scale of the individual across visits. The line is colored green if the clinical scale decreases between visits and red if the scale increases between visits. The blue line is the best-fit Gaussian process regression over the data (the blue shaded area represents the 95% confidence intervals of the fit). **d**, Plot of the KineDMD biomarker scale (solid blue line) along with the ethomic fingerprint-derived scores (lines flowing from the black dots) of the individuals with DMD from the KineDMD study as a function of age plotted on the *x* axis. **e**, Comparison of the KineDMD biomarker scale with an alternative quantification of disease progression in terms of actual muscle loss, as measured by a muscle MRI fat fraction of the vastus lateralis^23^ (the orange curve and shaded area represent the 95% confidence intervals based on data extracted from Naarding et al.^23^) on an age range of 0–25 years. Note that plot **d** is a zoomed in version of plot **e** for the age range 5–12 years.

The deficiencies of current criterion standard clinical measures (6MWD, NSAA, PUL) are further highlighted by the fact that DMD is a genetically based continuously progressing disease from fetal conception to death. Yet criterion standard clinical measures do not progress with age as the disease does; instead they can show transient improvement in motor function (Fig. 5a–c) since they are confounded by the children’s development and ‘improved’ motor performance up to the age of 7 years. This transient and apparent improvement is followed later by a rapid change induced by functional decline, such as loss of ambulation, which causes disproportionate functional loss in 6MWD or NSAA assessments. Also, we observed a vast variability across individuals for the criterion standard functional biomarkers (Fig. 5a–c shaded area). In addition, 6MWD or NSAA are motivation-dependent, which further limits their use as endpoints in clinical trials. Therefore, especially in the context of drug development, they are not ideal markers of the global disease, leading to artificial subdivision of therapeutic windows assessed in DMD clinical trials.

We developed an AI system for discovering a better biomarker among the infinitely many ways one could define one. Specifically, we used the Bayesian optimization methodology (Methods) to find a digital biomarker that would encapsulate biomedical invariants, which in a genetic disease such as DMD invariably progresses with age. At the same time, the rate of disease progression may vary over time. Therefore, we tasked the AI search to find a better biomarker, which is self-consistent with the observational data. Specifically, we constrained the Bayesian optimization to search for a monotonically increasing biomarker that starts at 0 at age 0 and reaches the maximum value of 1 at 25 years of age. Of all the many possible models satisfying these constraints, we defined the KineDMD ethomic biomarker as the model that can be best-fitted using the ethomic fingerprints. This AI search produced the new KineDMD ethomic biomarker, which progresses with age in an S-shaped sigmoid curve form and is entirely defined by daily-life data. The marker showed small variability across the individuals in our cohort (Fig. 5d compared to Fig. 5a–c). To validate our KineDMD ethomic biomarker, we compared its age dependence with an indirect quantification of disease progression, percentile curves derived from muscle MRI quantifying muscle loss by fat replacement^23^ (Fig. 5e). Interestingly, the exact mathematical shape of the KineDMD biomarker lies within error margins of the percentage fat fraction MRI data, even though at no point did we use information on the MRI curve in the development of our biomarker.

## Discussion

Advanced therapies such as gene therapies are rapidly progressing toward the clinic for degenerative and rare diseases. Yet, the methodology for assessing these new therapies in clinical trials, the clinical endpoints, have not kept up with the pace of progress and have remained largely unchanged for half a century (for example, the 6MWD test was developed almost 60 years ago^24^), and are in stark contrast to high-performance medicine^2^. Existing movement behavioral biomarkers for DMD reported in the literature^4^ are often digital in that they use digital sensors and are focused on single features for measuring disease, such as the mean number of steps walked^25,26^, stride length to height ratio^27^, hip kinetics during gait^28^, upper extremity reachable workspace^29^ and the 95th percentile stride velocity^30^ (which has now been validated as a secondary endpoint by the European Medicines Agency^31^). Unlike our daily-life settings, most of these measures are obtained in laboratory-based settings with the exception of ActiMyo^32^. Moreover, many of these movement behavioral markers are focused on lower-body performance, which precludes them from capturing DMD progression after loss of ambulation, unlike our ethomic biomarker. The ability of our KineDMD biomarker to match data spanning the whole lifetime disease trajectory suggests that we have distilled essential elements of the disease’s impact on daily-life movement behavior and these will also enable the study of patients with DMD across life-changing milestones, such as loss of ambulation. It is therefore plausible that such a biomarker can be used in clinical trials as an endpoint.

We are proposing a principled approach for using all-body measures that are obtained from holistic daily life-based assessment of patients in their normal life. This makes ethomic biomarkers uniquely reflective of the actual functional whole-body capacity of the individual. Hence, this principled allows us to predict disease trajectory accurately. This was a challenge historically because natural human movement behavior is highly variable and it is by eye difficult to spot an underlying simplicity that reflects on changes in disease mechanisms^5^. However, AI and machine learning have been leveraged to identify complex interactions and disease patterns directly from the clinical data generated, thereby avoiding observer-introduced biases^3,6,33^ and thus exceeding the performance of human experts^34^.

Our study has some limitations. Our cohort does not cover the full age range of 25 years used in the ethomic biomarker prediction. We are extrapolating beyond the age range of our population. Larger cohorts of individuals across different ambulatory status and application of the methodology in interventional trials will be essential to strengthen the points of acceptability, assessment of bias, validity and corroborate further the predictive capacity of our approach. Another limitation of our study is the use of the PUL scale, which is known to have a ceiling effect in the younger population. However, we selected the PUL scale in our study because it is the most widely used clinical scale for upper-limb assessment in clinical trials.

By combining an approach that embraces daily-life movement behavior with machine learning and ethomics, our AI biomarker provides a systematic pathway for determining when a new therapy effect occurs or weans off in real time. Focusing on natural movement behavior instead of reductionistic clinical assessments provides both greater coverage and a more robust measure of a patient’s motor capability. Moreover, in the context of drug development, AI and machine learning can be used to identify and characterize new disease features that are not driven by clinical biases and may reliably measure disease progression and potential response to new therapies. This ethomic approach promises to shorten the duration and cost of clinical trials but also allows to reduce the sample size required since patient recruitment is often a major challenge in developing drugs for rare or complex diseases. Our approach can be easily applied to other neuromuscular, neurodegenerative or acquired neurological diseases such as stroke and dementia but also in other areas of medicine wherever a patient’s disease state is reflected in how they are able to conduct their daily life, such as with heart or lung diseases. Thus, our work has the potential to accelerate the development of new therapies for many rare diseases and beyond, especially where progression is slow or discontinuous and difficult to detect.

## Methods

### Observational study information

The present study was conducted with approval from the appropriate research ethics committees and host institutions (ref. no. 18/SW/0012 South West—Cornwall & Plymouth Research Ethics Committee). It was conducted in full conformity with all applicable laws and regulations, including the International Conference on Harmonization Guidelines for Good Clinical Practice (CPMP/ICH/135/95) and relevant articles of the Declaration of Helsinki (seventh revision, 2013). The study protocol is provided in the Supplementary Note. All individuals were recruited between May 2018 and April 2019 and participated at Great Ormond Street Hospital, London, UK. Written informed consent was obtained from each study participant and their parents/legal guardians. Data from one individual’s visit could not be included in the analysis because his data files were corrupted. No other individuals were affected by this issue. One of the individuals refused to complete the 6MWD test on two of his visits because of behavioral issues. Two individuals refused to complete the 6MWD on one of their visits. Only the 6MWD data from those individuals were not included in the analysis and the rest of the data from those individuals were included. PUL data from one visit were not included for technical reasons. MyoGrip data were not recorded for two visits because of equipment issues. The study was registered at the UK DMD Hub website https://dmdhub.org/trials/kinedmd/. Additional data on the 6MWD, NSAA and PUL clinical scales from a larger cohort with DMD were provided by Ospedale Gemelli (institutional review board no. 16464/16 ID:1161, Fondazione Policlinico Gemelli Istituto di Ricovero e Cura a Carattere Scientifico). Informed consent was obtained from all participants of the Gemelli study.

### Participant characteristics

The clinical trial included two cohorts: male individuals with DMD and age-matched male healthy controls. Clinical diagnosis of DMD was genetically confirmed by MLPA, full gene sequencing or any other state-of-the-art diagnostic technique. At recruitment, 21 males with DMD with a mean age of 9.4 years (range: 6–17 years), 18 ambulant and 3 non-ambulant were included in this study. Furthermore, 17 age-matched healthy controls, mean age of 10.4 years (range: 4–16 years) were recruited. Individuals with DMD and healthy controls were followed over the course of 12 months and were examined at baseline, 6 and 12 months at Great Ormond Street Hospital (healthy controls at baseline and 12 months). A summary of baseline characteristics of the enrolled individuals is provided in Supplementary Table 1 for individuals with DMD and Supplementary Table 2 for healthy controls.

The Gemelli data included 88 male individuals with DMD (292 visits) with a mean age of 9.2 years (range: 4–24 years). Of the 88 individuals, 27 had only 1 visit. Of the remaining 61 individuals who had more than 1 visit, 44 had a visit in the same range as the 6 monthly visits of the KineDMD study. See Supplementary Table 3 for the characteristics of the entire Gemelli cohort.

### Assessments/procedures

The assessment of each individual followed standard physiotherapy and clinical assessment procedures at each hospital visit and followed the presented order. All assessments were carried out in and around Great Ormond Street Hospital: (1) recording of medical history and medication history; (2) height and weight measurement; (3) physical examination including vital signs; (4) fitting of motion capture suit, calibration and testing of wearing comfort (10 min); (4) motor functional and strength tests including the following: (a) NSAA; (b) 6MWD; (c) PUL scales for ambulant and non-ambulant individuals; (d) hand strength by MyoGrip.After step (4) and between assessments (step (5) a–d) or thereafter individuals had considerable time off ‘by design’. We set aside specifically these longer periods of unconstrained, free movement behavior as separate data collection periods. Individuals and their carers were given full freedom to engage in spontaneous behavior of everyday activities. While data collection was continuing without limiting the individuals, they could move freely and engage in activities such as visiting play areas indoors and outdoors. Specific activities were carried out by all males, including dressing (donning and doffing a coat), eating, drinking, playing on a gaming device, constructing something with Lego building blocks, reading a book, writing or coloring depending on age and ability, resting lying down to simulate sleeping and watching TV. For those who were able, walking along the corridors and going up and down the stairs were also completed. Participants were given free time to explore open spaces both indoors and outdoors to engage in activities they chose such as football, basketball or pool. Our cohort with DMD did not undergo any MRI scans on the day of the assessment because MRI is not considered standard of care for routine assessments in DMD.

### NSAA and timed test

The NSAA is a clinician-administered scale that rates an individual’s performance on various functional activities^18,19^. During this assessment, individuals are asked to perform 17 different functional activities including standing, walking, standing up from a chair, single-leg stance (right then left), ascending and descending a 15-cm high step, lift head while supine, lying to sitting, lying to standing, standing on heels, jumping, hopping on the right leg, hopping on the left leg. Patients will be graded as follows: 2 = normal, no obvious modification of activity; 1 = modified method but achieves goal independently of physical assistance from another; and 0 = unable to achieve goal independently.

### PUL

The PUL 2.0 is an outcome measure that has been developed and validated for use in late ambulant and non-ambulant patients with DMD^20^. PUL 2.0 has 22 items, which measure strength and function using specific tasks and are categorized into shoulder, elbow and distal domains. Each participant was assessed for the entry point of the PUL 2.0 using the modified Brooke test. The PUL 2.0 was then carried out using the standardized protocol and scoring criteria.

### 6MWD

The 6MWD is an assessment of ambulation using a modified version of the American Thoracic Society guidelines. The modified version has been used in many clinical trials in DMD as a primary endpoint for therapies such as eteplirsen, ataluren and microdystrophin gene transfer. The test requires the child to walk for 6 min along a taped 25-m course^21,22^.

The assessors in this study were experienced neuromuscular physiotherapists (supervised by V.S.), who were individually trained and assessed in the independent use of the sensor suit.

### MyoGrip

MyoGrip is a very sensitive myometer that evaluates hand grip strength^35–37^

MyoGrip data were collected for each participant using a three-trial maximal effort grip strength protocol. Raw data, collected in kg, were then analyzed using the percentage predicted^38^.

### Sensor suit protocol

On the day of the assessment, each participant and family were met at Great Ormond Street Hospital by the study physiotherapist and taken to the physiotherapy department or clinical research facility. Consent and assent were gained, and the family/participant shown the motion capture suit (see next subsection for details). Anthropometric data and a brief past medical history were obtained, including details of DMD diagnosis, current medication review and therapy input. Once the above had been gathered, the Xsens suit, which included 17 wireless inertial measurement unit (IMU) sensors, as mentioned in the protocol, was donned. The IMUs were placed as per the manufacturer’s guidelines at the head, shoulders, upper arms, forearms, hands, sternum, pelvis, thighs, shanks and feet.

Once calibration was complete, each participant was video-recorded to allow for visual data to be captured alongside Xsens motion data. At this point, the families had a discussion as to whether a parent would remain at the appointment or leave the child or a mix. However, if parents attended for the appointment, they were not allowed to interfere or assist with any assessment or data collection.

While wearing the suit and being video-recorded, each participant was asked to complete a series of physiotherapy assessment measures according to their ability: NSAA including timed tests of timed rise from floor and 10-m walk/run; PUL 2.0; 6MWD; and two different grip and pinch strengths using MyoTools (MyoGrip and MyoPinch).

After the formal assessments, there was a period, up to 2 h, where guided ADLs were recorded. Guided activities included walking, running, eating (participants were taken to the hospital canteen for lunch), ascending and descending stairs (if able), writing, reading, dressing (for example, putting on a coat), resting, playing computer games and Lego building.

Once the guided ADL had been completed, each participant was allowed up to 1 h of free play. This was directed by the participant and included activities such as playing basketball, football, gaming, pool. Individuals could choose their degree of activity when playing or engaging in snack or lunch activities. There was no maximum capability requirement or even encouragement. These activities were limited only by safety and requirement to stay within the hospital setting.

During the assessment period, any events such as falls or the need for recalibration were documented. Recalibration of the motion capture suit was carried out as and when required in accordance with the quality of data being collected and recorded. After the above recordings and data collection, the Xsens suit was removed, sensors cleaned and recharged for the next participant.

### Body sensor network ‘suit’ (wearable full-body motion tracking)

Individuals wore a wearable motion tracking suit of 17 wireless IMU sensors (Xsens MVN Awinda; Xsens Technologies B.V.) that recorded full-body kinematic data at 60 Hz. Data acquisition was controlled via the MVN Analyze graphical interface (Xsens Technologies B.V.). The Xsens sensors showed high accuracy^39,40^ and the Xsens MVN system has been used and validated in tracking real-world movement behavior in many sports including football^41^, horse-riding^42^ and snowboarding^43^. The detailed properties of the IMU sensors are available in the product documentation from the vendor^44^. Xsens MVN uses a 23-segment biomechanical model of the human body with 22 joints and proprietary algorithms to reconstruct a three-dimensional (3D) human pose^45,46^ from the raw sensor information. This sequence of body poses is represented as a multidimensional time series of various joint angles of 22 joints of the body and the relative 3D positions of 23 body segments. The suit sensor was streamed wirelessly from the body sensor network to a local laptop and stored in an encrypted manner. These data were then securely transferred to our secure offline data storage for holding, data curation and subsequent analysis.

The full-body kinematics data from the Xsens MVN Analyze software (2018; 2019 were exported as XML files and were analyzed using custom software written in MATLAB (R2019b; MathWorks). We smoothened the kinematics data from the MVN export files using a centered moving average technique with a window size of 21 before further processing.

Note, that the full-body kinematics were extracted in joint angles in three d.f. for each joint that followed the International Society of Biomechanics recommendations for Euler angle extractions of *X* (abduction/adduction), *Y* (internal/external rotation) and *Z* (flexion/extension). To be clear, this standard approach for full-body kinematics includes representing hinge joints of the body (such as the elbow), which have only one d.f. but still being represented as three Euler angles (that is, three numbers).

### Ethomic fingerprints

From the suit data, we extracted a range of ethomic fingerprints (movement behavioral biomarkers) that highlight the differences between individuals with DMD and healthy controls. A detailed description of each of these ethomic fingerprints is presented below.

### Workspace volume

Workspace volume can be described as the volume generated by the movements of the limbs in space. The idea of workspace volume is illustrated in Fig. 2a. Since our participants were not static and were dynamically moving in space, we modified the concept slightly to make the estimation of the workspace volume more robust. We fixed each individual’s trunk to a single reference point and adjusted the location of the rest of the joints with respect to the trunk. Using the joints’ 3D locations in space, we separated the space in a grid of 2 × 2 × 2 cm voxels and then calculated its occupancy density (that is, how often a body part is in that voxel). Therefore, we could visualize the workspace volumes of the body as shown in Fig. 2a, where the color of each voxel represents the occupancy frequency on a log_10_ scale (blue very low probability, red very high probability). Using the generated density space of joint positions, we calculated the workspace volume by counting the non-empty voxels and multiplying the result by the single voxel volume (that is, 8 cm^3^). We calculated the workspace volume for all 22 joints of the body (called full-body) and separately for the upper-body and lower-body joints. Applying this analysis across all individuals, we observed that patients with DMD needed significantly less space than controls (Fig. 2a, *P* < 0.001, Kruskal–Wallis one-way analysis of variance (ANOVA)).

### Hip orbit area

As in workspace volume calculations, we used the suit’s biomechanical model and fixed the pelvis to a fixed location. We then calculated the location of the hip with respect to the pelvis and calculated the area generated by the hip orbit on all three planes (Fig. 2b). Applying the calculations to all individuals, our results showed a statistically lower area covered by the patients with DMD than the controls (Fig. 2b, *P* < 0.05, Kruskal–Wallis one-way ANOVA). Post hoc confirmation showed that clinical workers could spot the qualitative difference in observations of the hip orbit ‘by eye’ during the trial.

### Extremities velocities

Using the 3D locations of the extremities (hands and feet) in space, as provided by the suit’s biomechanical model, we calculated the linear velocity in each of the three axes of the earth-fixed reference coordinate system. We then calculated the magnitude of the velocity by applying root-mean-square operation and then calculated the mean and variance of the magnitude signal for each extremity. A comparison between the mean (and variance) of the linear velocities of the extremities of the individuals with DMD and healthy controls is presented in Fig. 2c and Extended Data Fig. 4b.

### Average joint velocity

One of the fingerprints we extracted from the kinematic joint angle data was based on the average angular velocities of the joints. DMD causes progressive muscle weakness, which should be reflected into slower movement speeds. The results in Extended Data Fig. 2a support our observations that the velocities achieved by individuals with DMD are statistically slower than healthy controls based on a Kruskal–Wallis one-way ANOVA test (*P* < 0.05).

### Autocorrelation full-width at half-maximum of joint angle velocities

We explored the joints’ autocorrelation full-width at half-maximum (FWHM) as a measure to evaluate the similarity between joint movements across time. This was evaluated by first calculating the autocorrelation up to a 10-s lag. The output was a single bell-shaped curve centered around the 0-s lag. The FWHM is defined as the width of the bell-shaped curve at the point when it reaches a 0.5 autocorrelation value (half maximum). This metric has been previously used as an indication of how rapidly the hand joint kinematics change^47^. We applied the FWHM on the joints and the results are shown in Extended Data Fig. 2b. The individuals with DMD had a significantly higher autocorrelation FWHM than healthy controls (*P* < 0.05, Kruskal–Wallis one-way ANOVA), which indicated that the movements of the individuals with DMD were changing more slowly.

### Variability of the joint angle velocities

From our analysis, we consistently found that the individuals’ joint velocities were best described by a logistic probability distribution. Therefore, we applied a logistic distribution on all joints and compared the scale parameter (*σ*) across individuals with DMD and healthy controls as a measure of the variability of joint velocities. The results are shown in Extended Data Fig. 3a where individuals with DMD exhibited a significantly lower *σ* than healthy controls (*P* < 0.01, Kruskal–Wallis one-way ANOVA) suggesting that individuals with DMD used a much smaller range of velocities.

### Duty cycle of the joint angle velocities

The next fingerprint we explored in the kinematic data analysis was based on each joint’s duty cycle—what percentage of time did individuals use each individual joint. We applied our motion detection algorithm based on the empirical distributions of the joint velocities to detect the movements for each individual joint. Our motion detection algorithm is based on empirical thresholds estimated by computing a histogram (100 bins) evenly spaced over the data range and choosing the value for which data had a probability of less than a set threshold. The movement regions were then selected by finding the areas that exceed the threshold value. The algorithm was applied on each signal dimension independently and the results were combined afterwards using a logical disjunction operator.

A comparison of the duty cycles of the patients with DMD and the healthy controls are shown in Extended Data Fig. 3b. It can be clearly seen that the duty cycle of the healthy controls is significantly greater than that of the individuals with DMD (*P* < 0.05, Kruskal–Wallis one-way ANOVA), which also agrees with the general observation that individuals with DMD moved their joints less compared with healthy controls.

### Linear acceleration of the body segments

We calculated the magnitude of the linear acceleration by applying root-mean-square operation and then calculated the mean and variance of the magnitude signal for the different body segments. A comparison between the mean and variance of the linear velocities of the extremities of the individuals with DMD and healthy controls is presented in Extended Data Fig. 4c,d.

### Feature selection and model evaluation

We applied a Gaussian process regression algorithm^48^ to combine the ethomic fingerprints and find a mapping against the standard clinical scales. Gaussian process regression is a state-of-the-art method that applies a nonlinear regression and can capture the uncertainty in the presence of high variability in the data in a principled manner. For each of our regressions, we used a nested cross-validation procedure for feature selection and model evaluation (to avoid leakage of the test data during the feature selection process). The inner cross-validation loop was used for the feature selection and the outer cross-validation loop was used to evaluate the performance of the model. We used a leave-one-subject-out (LOSO) (leave the rows corresponding to all visits of an individual) for both the inner and outer cross-validation loops. We used a combined forward and backward wrapper feature selection approach to select the most optimal subset of features in the inner cross-validation loop.

For *s* number of individuals with each *v* visits, the data consists of *s* × *v* rows and the outer cross-validation splits the data into *s* folds (ensuring all the visits of an individual are in a single fold and each fold contains only the rows corresponding to the visits of a single individual). So, we have *s* training and test folds. For each of the *s* training folds, a combined forward and backward feature selection was done using the LOSO cross-validation error of the inner cross-validation loop as the objective function; thus, a subset of features were generated for each of the *s* training folds. The most frequent subset among the *s* subsets was selected as the optimal subset because the frequency of the subset of features is a measure of the robustness of the subset of selected features to changes in the training data. Finally, the overall performance of the GP regression was evaluated for the selected optimal subset of features using the outer cross-validation for the *s* test sets. This nested cross-validation approach ensured that the test data in each fold of the outer cross-validation loop was never used during feature selection in the inner cross-validation loop and therefore provides a reliable estimate of model performance. The hyperparameters of the Gaussian process were chosen based on the cross-validation error on the inner nested loop. The predicted values from all the test folds of the outer fold were aggregated and the aggregate root mean squared error (RMSE) and coefficient of determination (*R*^2^) was calculated and reported.

### Cross-sectional and longitudinal predictions

The cross-sectional predictions of the 6MWD and NSAA scores were done only for ambulant individuals. In longitudinal prediction comparisons, the longitudinal prediction of the conventional biomarkers (6MWD, NSAA and PUL) using the biomarkers themselves (for example, predicting NSAA at T + 6 months using NSAA at T + 0 months) was compared against the longitudinal prediction of the conventional biomarkers using our ethomic fingerprints (predicting NSAA at T + 6 months using ethomic fingerprints at T + 0 months). The longitudinal prediction of the conventional biomarkers (6MWD, NSAA and PUL) at T + 6 months using the biomarker themselves at T + 0 months was done on the combined cohort of KineDMD and Gemelli studies to give more prediction power to the conventional biomarkers. The longitudinal prediction of the conventional biomarkers (6MWD, NSAA and PUL) at T + 6 months using the ethomic fingerprints at T + 0 months was done on the KineDMD cohort.

For the longitudinal results (Fig. 4c,f,i), which show the performance of the predictions as a function of the number of individuals used to build the machine learning models, *nCk* (up to a maximum of 1,000) combinations (where *n* is the total number of individuals in the dataset and *k* is the number of individuals used to build the machine learning model) of the models were built for each *k* and the mean and s.d. of the aggregate performance of the *nCk* models was reported.

### KineDMD ethomic biomarker modeling using Bayesian optimization

We chose hyperbolic tangent functions to model the KineDMD ethomic biomarker because the family of hyperbolic tangent functions can exhibit different monotonically increasing behaviors (such as linear, exponentially increasing with differing slopes, S-shaped sigmoid curves) for different parameter values. We used the following equation to model the KineDMD ethomic biomarker *Y* as a monotonically increasing function of age:

$${{{{Y}}}}\left( {{{{\mathrm{age}}}}} \right) = \left( {\left( {{{{\mathrm{tanh}}}}\left( {{{{X}}}} \right) - {{{\mathrm{tanh}}}}\left( {{{{{X\mathrm{min}}}}}} \right)} \right)/\left( {{{{\mathrm{tanh}}}}\left( {{{{{X\mathrm{max}}}}}} \right) - {{{\mathrm{tanh}}}}\left( {{{{{X\mathrm{min}}}}}} \right)} \right)} \right.$$

where *X* = *α* × age – *β*; *X*_min_ = *X*_(0)_ and *X*_max_ = *X*_(25)_; 0 ≤ *α* ≤ 0.5 and 0 ≤ *β* ≤ 5

The equation constrains the biomarker to be 0 at age 0 and monotonically increases and reaches the maximum value of 1 at 25 years of age. For different values of *α* and *β*, the equation generates different monotonically increasing behaviors such as linear, exponentially increasing with differing slopes, S-shaped sigmoid curves and so on. Of all the possible models of biomarker, we wanted to find the model that can be best-fitted using the ethomic fingerprints. We used Bayesian optimization^49,50^ to search the parameter space of *α* and *β* and find the best-fit model. The objective function used in the Bayesian optimization algorithm is the LOSO cross-validation regression error while regressing the biomarker using the ethomic fingerprints with the GP regression algorithm with feature selection. The acquisition function was set to expected improvement and a Gaussian process was used as the surrogate function. Additional constraint on the objective function was placed so that the biomarker was clinically meaningful. The ranges for the constraints of the ethomic biomarker disease scale were chosen based on our clinical experience. The disease scale should start at 0 at age 0 and reach 1 at age 25. The constraint for the range of the ethomic biomarker at age 5 was chosen to be between 0.01 and 0.15 because some patients are not even overtly symptomatic (corresponding to 0.01 of the scale) at 5 years and others show the first signs of the disease (0.15). The constraint for the range of the biomarker at age 15 was chosen to be between 0.5 and 0.8 because patients lose lower-body ambulation (corresponding to 0.5 of the scale) and start losing upper-body ambulation and developing heart and respiratory problems (0.8) by age 15. Thus, the constraints were chosen to be clinically meaningful and large enough to allow a wide range of functions.

### Rights retention statement

For the purpose of open access, the authors have applied a Creative Commons Attribution (CC BY) license to any author-accepted manuscript version arising from this submission.

### Reporting summary

Further information on research design is available in the Nature Research Reporting Summary linked to this article.

## Online content

Any methods, additional references, Nature Research reporting summaries, source data, extended data, supplementary information, acknowledgements, peer review information; details of author contributions and competing interests; and statements of data and code availability are available at 10.1038/s41591-022-02045-1.

## Supplementary information

Supplementary Information Supplementary Tables 1–5, Figs. 1–5 and Note (Study Protocol).

Reporting Summary

## Data Availability

The data used in the study are not publicly available because they contain information that could compromise research participant privacy/consent. Anonymized data can be made available upon reasonable request for academic purposes.

## Code availability

The machine learning code that supports the findings of this study is available at 10.6084/m9.figshare.20102678.
