# Supplementary material for: Wearable full-body motion tracking of activities of daily living predicts disease trajectory in Duchenne muscular dystrophy
Source: Nat Med. 2023 Jan 19;29(1):95–103. doi: 10.1038/s41591-022-02045-1 (PMC9873561; doi:10.1038/s41591-022-02045-1)
Supplement: Supplementary file 1 — Supplementary Tables 1–5, Figs. 1–5 and Note (Study Protocol). [file 41591_2022_2045_MOESM1_ESM.pdf]

nature medicine

Article https://doi.org/10.1038/s41591-022-02045-1

Wearable full-body motion tracking of activities of daily living predicts disease trajectory in Duchenne muscular dystrophy

In the format provided by the authors and unedited

List of supplementary tables

Supplementary Table 1. Characteristics of DMD subjects in the KineDMD study

**KineDMD DMD subjects**	**Visit 1**	**Visit 2**	**Visit 3**
N	21	14	11
Ambulant: non ambulant	18:3	13:1	10:1
Mean Age years (range)	9.43<br>(6.2 – 16.9)	9.35<br>(6.72 -11.21)	9.77<br>(7.2 – 11.75)
Mean height, cm (range)	129.0<br>(110-152)	130.8<br>(112.5 – 149)	132.3<br>(117.0 -149.0)
Mean weight, kg (range)	32.6<br>(19-65)	31.78<br>(20.20 – 59.3)	29.0<br>(21.0 - 41.0)
Steroids<br>(Pred: Def)<br>(Daily: 10/10)	21<br>7:14<br>12:9	14<br>3:11<br>10:3 (1 alt)	10<br>2:8<br>7:3 (1 alt)
NSAA (of ambulant population)	24 (3-34)	24 (14-33)	22 (11-33)
6MWD (of ambulant population)	319 (125-475)	353 (187-468)	357 (158-478)
PUL	38 (22 – 42)	40 (35-42)	39 (32-42)
PUL Upper	10 (0-12)	11 (9-12)	11 (7-12)
PUL Mid	16 (9-17)	17 (15-17)	17 (16-17)
PUL Distal	12 (10-13)	12 (11-13)	12 (9-13)

Supplementary Table 2 Characteristics of healthy control subjects in the KineDMD study

**KineDMD HC subjects**	**V1**	**V2**
N	17	4
Mean Age, years (range)	10.34 (4.10 – 16.25)	9.9 (5.3 – 13.5)
Mean height, cm (range)	145.65 (113 – 189)	147 (121 – 173)
Mean weight, kg (range)	42.1 (18 – 70.6)	37.2 (21.5 – 53.3)
NSAA	34	34
6MWD	540 (325 -640)	576 (465 – 625)
PUL	42	42

Supplementary Table 3 Characteristics of DMD subjects in the Gemelli study

**Gemelli data**	
Number of subjects (Number of subjects with six monthly visits)	88 (44)
Number of visits (Number of six-monthly visits)	292 (122)
Ambulant: non-ambulant subjects	82:6
Mean Age years (range)	9.16 (3.68 – 24.72)

NSAA (of ambulant population)	23 (1-34)
6MWD (of ambulant population)	393 (87 -605)
PUL	39 (14-42)
PUL Upper	10 (0-12)
PUL Mid	16 (5-17)
PUL Distal	12 (9-13)

Supplementary Table 4 List of the 66 joint angles measured by the motion capture suit, where the ordering of these joint angles corresponds to the order of the entries in the correlation matrices of Fig. 1.c.

L5S1 abduction
L5S1 rotation
L5S1 flexion
L4L3 abduction
L4L3 rotation
L4L3 flexion
L1T12 abduction
L1T12 rotation
L1T12 flexion
T9T8 abduction
T9T8 rotation
T9T8 flexion
T1C7 abduction
T1C7 rotation
T1C7 flexion
C1 head abduction
C1 head rotation
C1 head flexion
Right T4 shoulder abduction
Right T4 shoulder rotation
Right T4 shoulder flexion
Right shoulder abduction
Right shoulder rotation
Right shoulder flexion

Right elbow abduction
Right elbow rotation
Right elbow flexion
Right wrist abduction
Right wrist rotation
Right wrist flexion
Left T4 shoulder abduction
Left T4 shoulder rotation
Left T4 shoulder flexion
Left shoulder abduction
Left shoulder rotation
Left shoulder flexion
Left elbow abduction
Left elbow rotation
Left elbow flexion
Left wrist abduction
Left wrist rotation
Left wrist flexion
Right hip abduction
Right hip rotation
Right hip flexion
Right knee abduction
Right knee rotation
Right knee flexion
Right ankle abduction
Right ankle rotation
Right ankle flexion
Right ballfoot abduction
Right ballfoot rotation
Right ballfoot flexion
Left hip abduction
Left hip rotation
Left hip flexion
Left knee abduction
Left knee rotation
Left knee flexion
Left ankle abduction
Left ankle rotation
Left ankle flexion
Left ballfoot abduction
Left ballfoot rotation
Left ballfoot flexion

Supplementary Table 5 List of features in the set of full body ethomic fingerprints along with their mean and standard error values for the DMD and healthy control (HC) cohorts and p-value of the Kruskal-Wallis one-way ANOVA test between the cohorts. Data are presented as mean (standard error).

Feature name	DMD Mean (SE) n=46 visits	HC Mean (SE) n=21 visits	p-value
Workspace volume - full body joints	0.63(0.02)	0.87(0.05)	2.84E-05
Workspace volume - lower body joints	0.37(0.02)	0.53(0.03)	0.000139476
Workspace volume - upper body joints	0.29(0.01)	0.38(0.02)	0.000147511
Hip orbit area - coronal plane	0.03(0.004)	0.04(0.01)	0.0498835
Hip orbit area - sagittal plane	0.03(0.004)	0.04(0.01)	0.0186555
Hip orbit area - transverse plane	0.05(0.003)	0.06(0.004)	0.0142659
Average velocity - left foot	0.11(0.01)	0.19(0.01)	7.03E-06
Average velocity - right foot	0.11(0.01)	0.19(0.01)	1.04E-05
Average velocity - left hand	0.10(0.01)	0.14(0.01)	0.00177556
Average velocity - right hand	0.11(0.004)	0.14(0.01)	0.000394114
Variance of velocity - left foot	0.07(0.005)	0.16(0.02)	1.69E-07
Variance of velocity - right foot	0.07(0.005)	0.16(0.02)	1.57E-07
Variance of velocity - left hand	0.05(0.01)	0.08(0.02)	0.00135868
Variance of velocity - right hand	0.04(0.003)	0.09(0.02)	3.33E-05
Average velocity - left ankle flexion	10.88(0.64)	14.06(0.8)	0.0130581
Average velocity - left elbow flexion	19.61(0.9)	24.58(1.35)	0.00620469
Average velocity - left hip flexion	6.31(0.37)	7.97(0.56)	0.0267691
Average velocity - left knee flexion	15.85(1.33)	22.88(1.39)	0.00206688
Average velocity - right ankle flexion	10.52(0.62)	14.51(0.9)	0.00166601
Average velocity - right elbow flexion	19.70(0.81)	25.12(1.47)	0.00225067
Average velocity - right elbow rotation	18.60(0.82)	24.12(1.25)	0.000272076
Average velocity - right hip flexion	13.61(0.91)	18.54(1.16)	0.00435374
Average velocity - right knee flexion	16.49(1.31)	22.83(1.35)	0.00490606
Autocorrelation FWHM - C1-head abduction	406.56(5.24)	372.95(6.92)	0.000646285
Autocorrelation FWHM - C1-head flexion	412.71(8.6)	382.41(10.08)	0.0031454
Autocorrelation FWHM - C1-head rotation	429.09(8.25)	406.27(11.51)	0.0250697
Autocorrelation FWHM - L5-S1 rotation	342.17(9.25)	322.35(7.47)	0.0167077

Autocorrelation FWHM - left ankle flexion	276.36(4.75)	242.9(4.29)	2.01E-05
Autocorrelation FWHM - left elbow abduction	351.81(4.39)	326.25(4.45)	0.000616757
Autocorrelation FWHM - left knee flexion	282.12(3.33)	257.63(2.78)	2.99E-06
Autocorrelation FWHM - left wrist abduction	339.44(3.23)	323.6(4.08)	0.00435374
Autocorrelation FWHM - right ankle flexion	273.08(4.97)	236.29(4.76)	1.06E-05
Autocorrelation FWHM - right elbow abduction	350.17(4.56)	324.65(5.63)	0.00133782
Autocorrelation FWHM - right elbow flexion	338.04(5.98)	318.39(6.16)	0.0357019
Autocorrelation FWHM - right elbow rotation	302.26(6.78)	275.75(7.38)	0.0121541
Autocorrelation FWHM - right knee flexion	286.05(3.15)	257.34(3.21)	2.90E-07
Autocorrelation FWHM - right shoulder abduction	361.44(4.61)	334.08(5.32)	0.000421936
Autocorrelation FWHM - right wrist abduction	340.30(3.48)	326.17(4.25)	0.0161368
Autocorrelation FWHM - T4-right shoulder abduction	346.90(6.36)	307.28(12.13)	0.00418256
Autocorrelation FWHM - T4-right shoulder flexion	313.11(9.06)	279.54(10.84)	0.00370502
Duty cycle - C1-head flexion	0.53(0.01)	0.59(0.02)	0.0324767
Duty cycle - C1-head rotation	0.56(0.01)	0.62(0.02)	0.0105108
Duty cycle - left ankle abduction	0.32(0.02)	0.47(0.02)	5.17E-05
Duty cycle - left hip abduction	0.31(0.02)	0.45(0.02)	0.000561429
Duty cycle - left knee flexion	0.32(0.02)	0.48(0.03)	6.08E-05
Duty cycle - right ankle abduction	0.34(0.02)	0.47(0.02)	0.000234408
Duty cycle - right elbow abduction	0.54(0.02)	0.61(0.02)	0.0219493
Duty cycle - right elbow flexion	0.63(0.02)	0.7(0.02)	0.0145257
Duty cycle - right elbow rotation	0.70(0.01)	0.79(0.02)	0.000616757
Duty cycle - right hip abduction	0.31(0.02)	0.45(0.02)	0.000535533
Duty cycle - right knee flexion	0.33(0.02)	0.49(0.03)	0.000191757
Duty cycle - right shoulder abduction	0.47(0.02)	0.55(0.03)	0.028567
Duty cycle - right shoulder flexion	0.56(0.02)	0.64(0.03)	0.0198362
Duty cycle - right shoulder rotation	0.59(0.02)	0.67(0.02)	0.0242556
Duty cycle - right wrist flexion	0.70(0.01)	0.75(0.02)	0.0276554
Correlation between C1-Head abduction and left elbow flexion	0.01(0.005)	-0.03(0.01)	9.31E-05
Correlation between C1-Head abduction and left shoulder rotation	0.00(0.01)	-0.06(0.02)	0.00117068

Correlation between C1-head abduction and right shoulder abduction	-0.03(0.01)	0.02(0.01)	8.87E-05
Correlation between C1-head flexion and T4-right shoulder flexion	-0.24(0.02)	-0.14(0.02)	0.00135868
Correlation between C1-head rotation and right shoulder rotation	-0.03(0.01)	-0.07(0.01)	0.002817
Correlation between L5-S1 rotation and left hip flexion	0.30(0.03)	0.47(0.03)	0.000588488
Correlation between right ankle abduction and left knee flexion	0.36(0.03)	0.29(0.02)	0.00941124
Correlation between right hip abduction and left knee flexion	-0.34(0.03)	-0.26(0.03)	0.011306
Correlation between right hip abduction and right knee flexion	0.48(0.03)	0.37(0.03)	0.0012798
Correlation between right hip flexion and left ball foot flexion	-0.06(0.02)	-0.2(0.02)	2.33E-06
Correlation between right knee flexion and left knee flexion	-0.38(0.03)	-0.25(0.02)	0.000487045
Correlation between right shoulder abduction and right wrist abduction	-0.02(0.01)	0.004(0.01)	0.0180156
Correlation between right shoulder flexion and right wrist flexion	0.00(0.01)	-0.06(0.01)	0.000359416
Correlation between right shoulder rotation and right wrist rotation	0.03(0.01)	-0.01(0.01)	0.000236598
Correlation between T4-right shoulder rotation and right elbow rotation	-0.05(0.01)	-0.01(0.01)	0.00235719
Variability of joint velocity - L5S1 rotation	2.19(0.11)	3.23(0.18)	1.42E-05
Variability of joint velocity - left elbow flexion	15.74(0.71)	19.8(1.05)	0.00301811
Variability of joint velocity - left hip flexion	11.38(0.8)	15.89(0.84)	0.00122412
Variability of joint velocity - left knee flexion	13.95(1.16)	19.67(1.08)	0.00206688
Variability of joint velocity - right elbow flexion	15.79(0.63)	20.13(1.13)	0.00152673
Variability of joint velocity - right elbow rotation	14.64(0.64)	18.88(0.95)	0.000234408
Variability of joint velocity - right hip flexion	11.34(0.79)	15.51(0.9)	0.00301811
Variability of joint velocity - right knee flexion	14.47(1.13)	19.61(1.04)	0.00596877
Average acceleration - left foot	0.95(0.07)	1.62(0.08)	4.38E-06
Average acceleration - neck	0.44(0.02)	0.75(0.06)	2.56E-06
Average acceleration - right foot	0.97(0.07)	1.64(0.08)	2.33E-06

Average acceleration - right forearm	0.95(0.04)	1.28(0.09)	0.000973653
Average acceleration - right hand	1.07(0.05)	1.44(0.09)	0.000512849
Average acceleration - right shoulder	0.67(0.04)	0.92(0.08)	0.0102276
Average acceleration - right upper arm	0.58(0.03)	0.89(0.07)	3.75E-05
Variance of acceleration - left foot	7.76(0.9)	17.59(1.18)	4.28E-07
Variance of acceleration - right foot	8.15(0.91)	18.58(1.25)	2.39E-07

List of supplementary figures

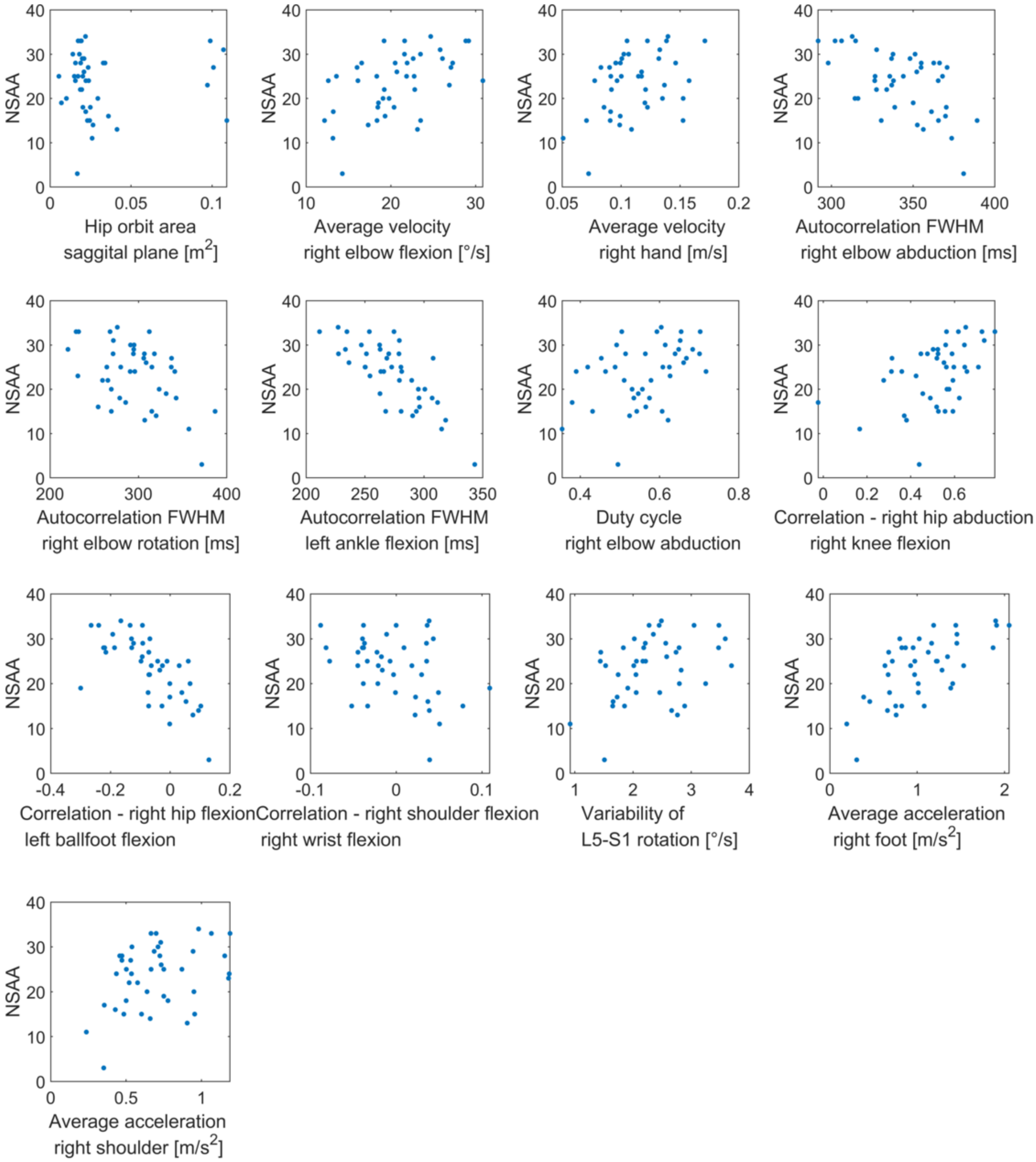

Supplementary Fig. 1 | Fingerprints selected by feature selection algorithm for cross-sectional prediction of NSAA. Scatter plot of the selected ethomic fingerprints against NSAA.

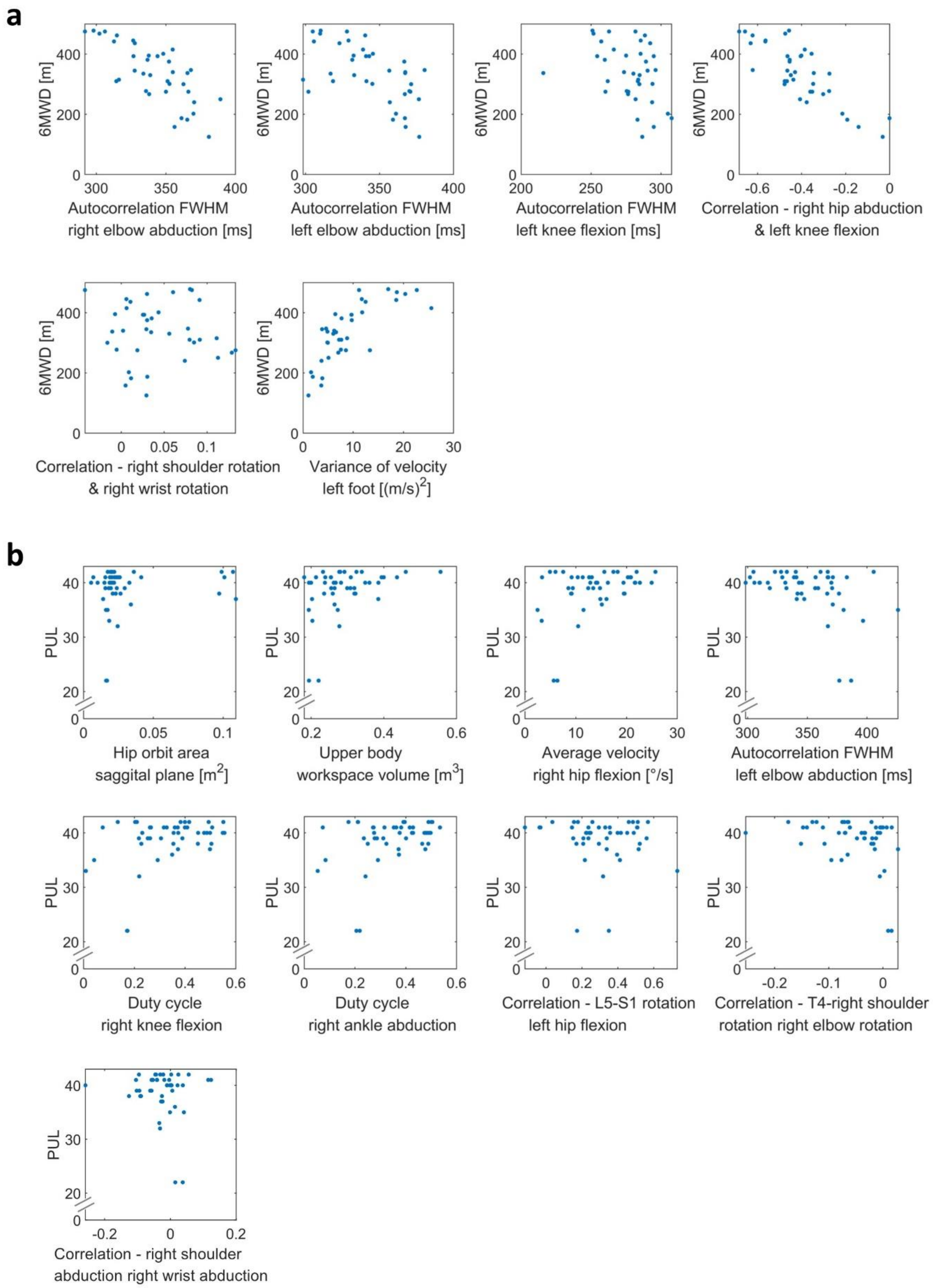

Supplementary Fig. 2 | Fingerprints selected by feature selection algorithm for cross-sectional prediction of 6MWD and PUL. Scatter plot of the selected ethomic fingerprints against 6MWD (**a**) and PUL (**b**).

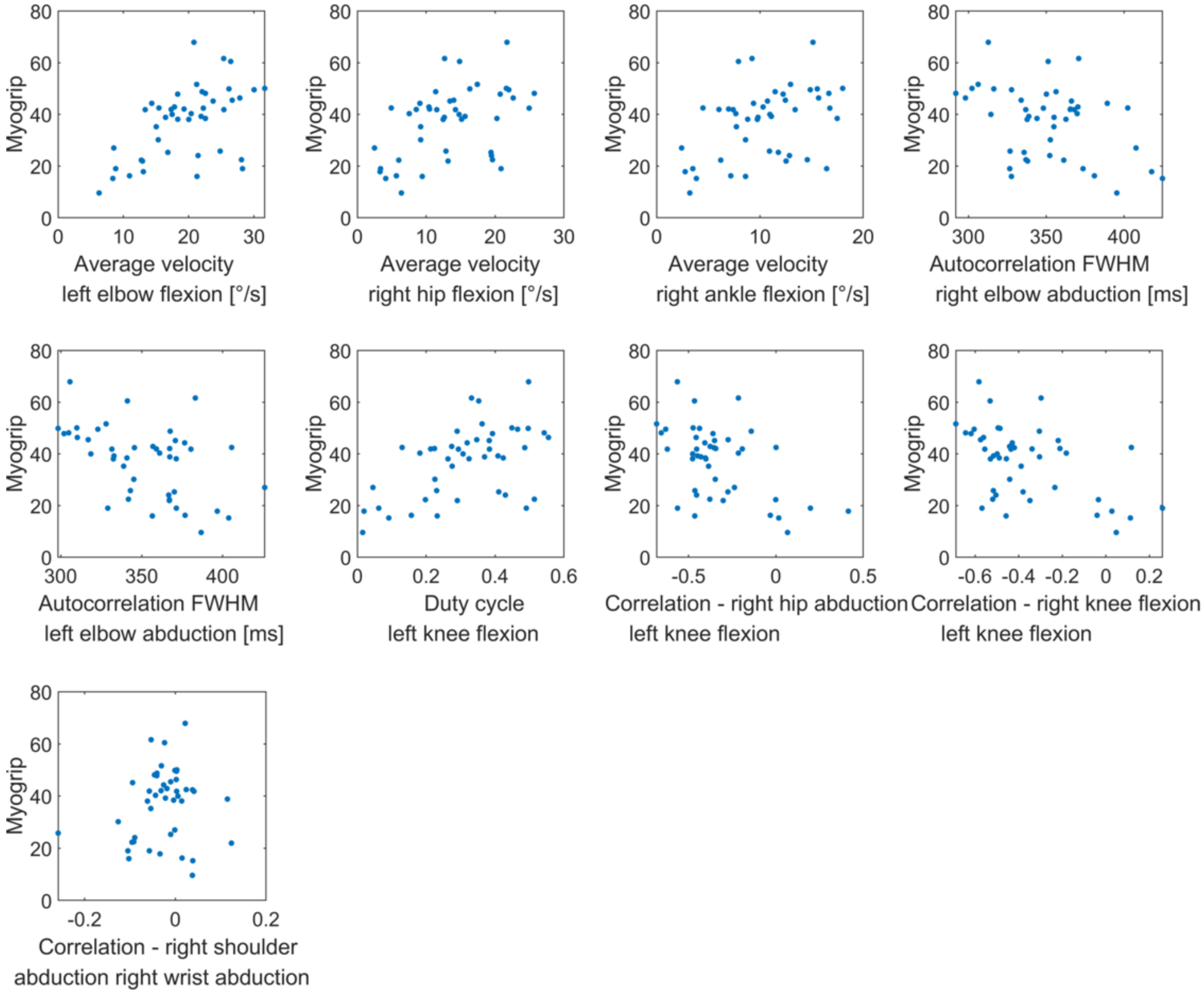

Supplementary Fig. 3 | Fingerprints selected by feature selection algorithm for cross-sectional prediction of Myogrip. Scatter plot of the selected ethomic fingerprints against Myogrip.

a

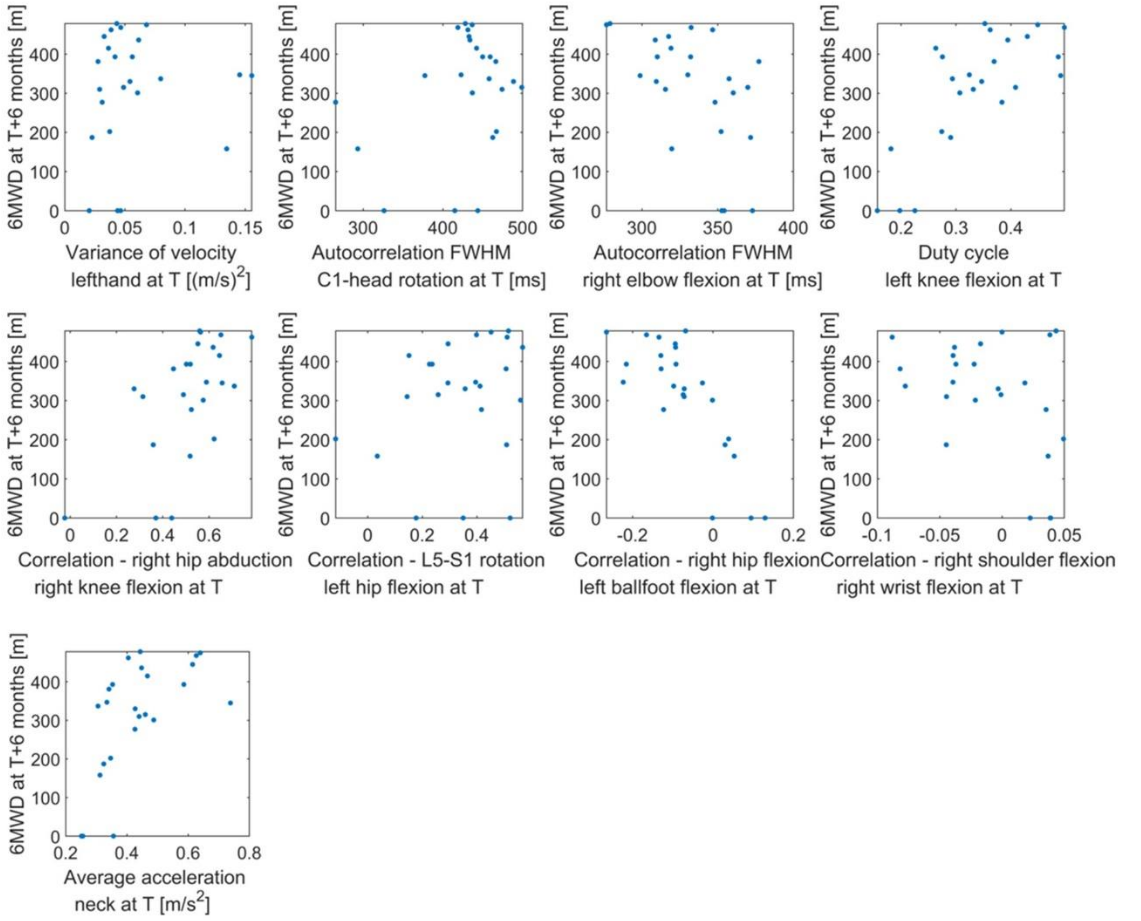

b

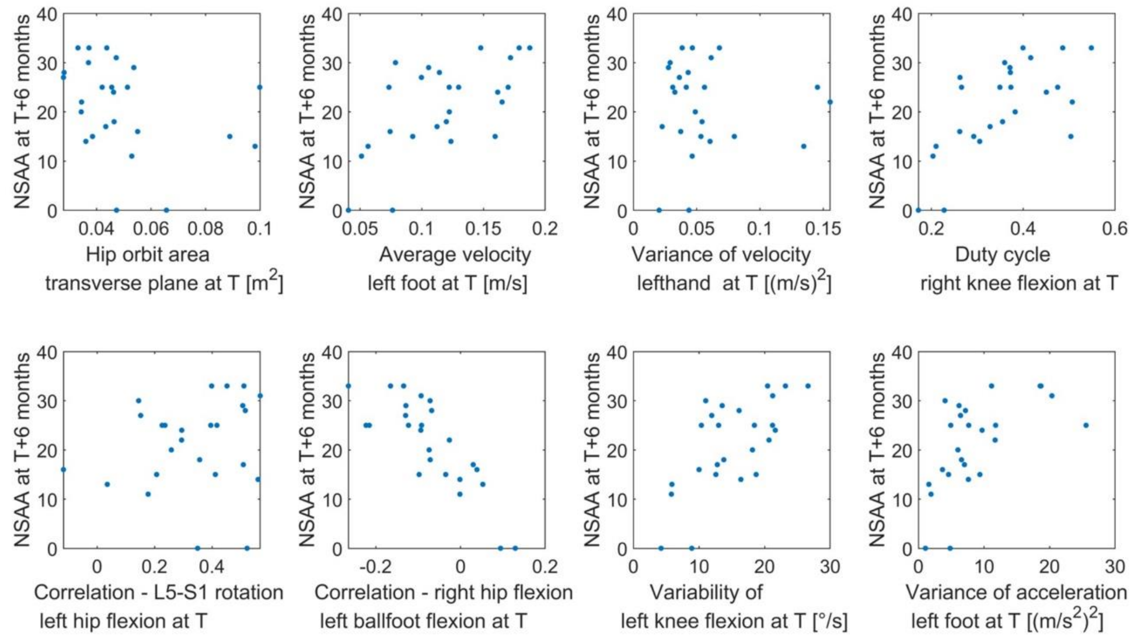

Supplementary Fig. 4 | Fingerprints selected by feature selection algorithm for longitudinal predictions of 6MWD and NSAA. Scatter plot of the selected ethomic

fingerprints from visit at T against 6MWD at visit T+6 months (**a**) and NSAA at visit T+6 months (**b**).

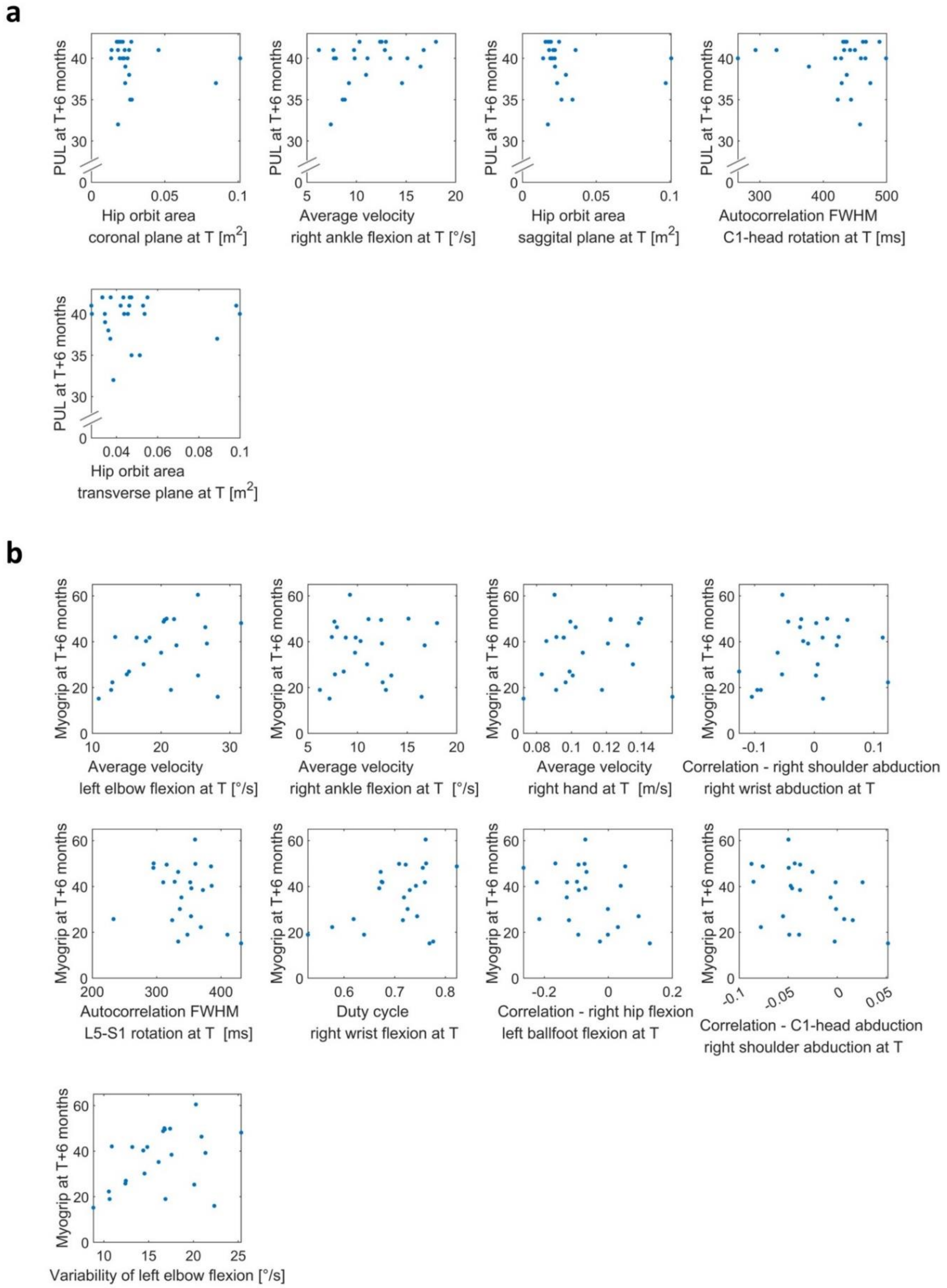

Supplementary Fig. 5 | Fingerprints selected by feature selection algorithm for longitudinal predictions of PUL and Myogrip. Scatter plot of the selected ethomic fingerprints from visit at T against PUL from visit at T+6 months (**a**) and Myogrip at T+6 months (**b**).

Supplemental Note – Study Protocol

CLINICAL INVESTIGATIONAL PLAN

KINEDMD: A study of kinematics in Duchenne Muscular Dystrophy

Version Number: 1.3
Date: 21st August 2019

Funder: Duchenne Research Fund

Chief Investigator: Assistant Professor Aldo Faisal
Dept. of Computing & Dept. of Bioengineering
South Kensington Campus, Imperial College London,
London, SW7 2AZ, UK

Clinical Principal Investigator: Professor Thomas Voit
Dubowitz Neuromuscular Centre
6st Floor, UCL Institute of Child Health
30 Guilford St,
London, WC1N 1EH, UK

Table of Contents

SIGNATURE PAGE 18
LIST OF ABBREVIATIONS 1
CONTACT DETAILS 2
SYNOPSYS OF THE CLINICAL INVESTIGATIONAL PLAN 3
INTRODUCTION 3
BACKGROUND AND SCIENTIFIC RATIONAL 5
SPECIFIC AIMS 7
DESIGN AND METHODOLOGY 7
STUDY DESIGN 7
INCLUSION CRITERIA FOR DMD SUBJECTS 8
EXCLUSION CRITERIA FOR DMD SUBJECTS 8
RECRUITMENT AND INFORMED CONSENT 8
DMD SUBJECTS' ENROLMENT 8
HEALTHY SUBJECTS' ENROLMENT 9
INFORMED CONSENT 9
STUDY TIMELINE 9
STUDY PROCEDURES 9
STUDY PROCEDURES FOR HEALTHY CONTROLS 9
Schedule of Events for healthy controls 10
STUDY PROCEDURES FOR DMD 11
Measurement of joint end range 11
Brooke scale for upper extremity 11
Performance of the Upper Limb (v2.0) 12
Myoset 12
North Star Ambulatory Assessment and timed tests 12
6-Minute Walk Test 12
Patient-reported outcome and quality of life questionnaire 12
User Experience Questionnaire 12
Bracelets (Apple Watch) home recording during daily activities 12
Suit system in clinic recording 13

Laboratory assessments for Biobank storage 13
Schedule of Events for DMD 13
Training 14
DATA MANAGEMENT 14
DATA CONFIDENTIALITY 15
RECORD KEEPING AND ARCHIVING 15
DATA COLLECTION AND COMPLETION OF CASE REPORT FORMS 15
RETENTION OF DOCUMENTATION 16
BIOSTATISTICS ANALYSIS 16
GENERAL PRACTITIONER INVOLVEMENT 17
POTENTIAL RISKS FOR PARTICIPANTS 17
POTENTIAL BENEFITS FOR PARTICIPANTS 17
SAFETY MONITORING PLAN 18
ADVERSE EVENTS 18
SEVERITY 19
CAUSALITY 19
EXPECTEDNESS 19
REPORTING OF ALL ADVERSE DEVICE EFFECTS 20
DURATION OF EXCLUSION 20
MANAGEMENT OF STUDY DROP-OUTS 20
DEFINITION OF END OF TRIAL 20
SUSPENSION OR PREMATURE TERMINATION OF THE CLINICAL INVESTIGATION 20
EARLY TERMINATION OF THE CLINICAL INVESTIGATION 21
DEVIATIONS FROM CLINICAL INVESTIGATION PLAN 21
PROCEDURES FOR RECORDING, REPORTING AND ANALYSING CIP DEVIATIONS 21
ETHICAL ISSUES 21
INDEMNITY 21
SPONSOR 22
DISSEMINATION OF RESULTS 22
REFERENCES 22

APPENDIX 25

USER EXPERIENCE QUESTIONNAIRE 26

PODCI 27

SOP FOR PLASMA/URINE COLLECTION FOR BIOBANK 29

SIGNATURE PAGE

The clinical study will be conducted in compliance with the protocol, GCP and the applicable regulatory requirement(s).

Principal Investigator:

Name of Principal Investigator Signature

Date:

On behalf of the Funding body, DRF:

Name of DRF representative Signature

Date:

List of Abbreviations

10-mt run	10 meters run/walk test
6-MWT	6 Minute Walk Test
ADE	Adverse Device Effect
ADL	Activities of Daily Living
AE	Adverse Event
ATS	American Thoracic Society
BP	Blood Pressure
CI	Chief Investigator
CRF	Clinical Research Facility
DMD	Duchenne Muscular Dystrophy
DNA	Deoxyribonucleic acid
FVC	Forced Vital Capacity
GCP	Good Clinical Practice
GOSH	Great Ormond Street Hospital
HC	Healthy Control
ICL	Imperial College London
IMU	Inertial Measurement Unit
JRO	Joint Research Office
MHRA	Medicines HealthCare Regulatory Agency
MLPA	Multiplex Ligation-dependent Probe Amplification
NSAA	NorthStar Ambulatory Assessment
PI	Principal Investigator
PODCI	The Pediatric Outcomes Data Collection Instrument
PUL	Performance of the Upper Limb
SADE	Serious Adverse Device Effect
SAE	Serious Adverse Event
TRFF	Time rise from floor from supine
UCL	University College London
UK	United Kingdom

Contact details

**Sponsor's representative**	Ruth Nicholson<br>Research Governance Manager<br>Room 221<br>Level 2, Medical School Building<br>Norfolk Place,<br>London W2 1PG<br>Email: r.nicholson@imperial.ac.uk<br>Tel: 020 7594 1862
**Chief Investigator (CI)**	Assistant Professor Aldo Faisal<br>Dept. of Computing & Dept. of Bioengineering<br>South Kensington Campus, Imperial College London,<br>London, SW7 2AZ, UK e-mail:<br>a.faisal@imperial.ac.uk
**Clinical Principal Investigator (PI)**	Professor Thomas Voit<br>UCL& GOSH Institute of Child Health<br>6th Floor, 30 Guilford St,<br>London, WC1N 1EH, UK<br>Tel: 02079052211 e-mail:<br>t.voit@ucl.ac.uk
**Research Physiotherapist**	Vic Selby<br>Great Ormond Street Hospital<br>Great Ormond Street London,<br>WC1N 3JH<br>e-mail: victoria.selby.12@ucl.ac.uk
**Statistician**	Deborah Ridout<br>ICH Pop, Policy & Practice Prog<br>Faculty of Pop Health Sciences<br>UCL GOS Institute of Child Health<br>e-mail: d.ridout@ucl.ac.uk tel:<br>0207 905 2706
**Funding body**	Duchenne Research Fund<br>Sheli Rodney<br>Director of Operations<br>Duchenne Research Fund<br>www.duchenne.org.uk email:<br>sheli@duchenne.org.uk

Synopsys of the clinical Investigational Plan

**Title:**	KINEDMD: a study of kinematics in Duchenne Muscular Dystrophy
**Devices:**	Apple Watches and ETHO sensor-suit system
**Phase of Investigation:**	Proof of concept
**Objectives:**	To investigate kinetic behaviour in subjects affected by Duchenne Muscular Dystrophy (DMD)
**Investigation design** in **and methods:**	Longitudinal study will explore kinetic behavioural biomarkers in DMD subjects using wearable sensors
**Investigation duration per participant:**	Each DMD participant will be attending 3 study visits at Great Ormond Street Hospital over 12 months and will be followed up by phone in between visits.<br>Healthy volunteers will be attending 2 study visit and will be followed up by phone for 2 weeks
**Estimated total Investigation duration:**	24 months
**Planned Investigation sites:**	Great Ormond Street Hospital Imperial College London
**Total number of participants planned:**	Up to 30 participants with confirmed DMD diagnosis (26ambulant and 4 non-ambulant)<br>Up to 30 age/gender matched healthy controls
**Main inclusion/exclusion criteria:**	DMD boys aged 5 - 18 years who have a confirmed diagnosis of DMD with a Brooke score □4 (non-ambulant); 6MWT 280- 450 meters, NSAA total score □20, time rise from supine □ 10 seconds (for ambulant) and no symptomatic cardiomyopathy, severe scoliosis or significant neuro-cognitive impairment.
**Statistical methodology and analysis:**	All data will be analysed with Stata. Prior to statistical analyses, all data per outcome measure will be checked for normal distribution by visual inspection, histogram plots. Descriptive Statistical methods will be applied.

Introduction

Duchenne muscular Dystrophy (DMD, OMIM 310200) is in an X-linked recessive muscle disease of childhood with a global prevalence of 1.7-4.2 per 100,000 (Theadom et al., 2014) and average global incidence of approximately 1:5000 live males. (Ellis et al., 2013) DMD is caused by mutations in the dystrophin-encoding *DMD* gene. Lack of dystrophin products results in progressive muscle weakness, leading to loss of ambulation and premature death secondary to cardiac and respiratory complications. At present, there is no curative treatment.

Affected children are diagnosed between 4 and 5 years of age, (Bushby et al., 1999) with some children being diagnosed as late as 9 years of age, figure which has changed little over the last 3 decades. The classic clinical presentation includes the following: delayed gross motor milestones, abnormal gait, calf hypertrophy, frequent falls and muscle cramps. These features may present in isolation or in combination with language delay, intellectual disability, and/or behavioural problems. (Emery, 2002)

Following an initial period of apparent stability in relation to muscle weakness, as they become older, DMD boys become progressively weak. Muscle weakness is more pronounced in the hip extensors, as a result the pelvis becomes unstable. Contractures also develop, most commonly affecting the Achilles tendons and the iliotibial bands. The disease evolves into muscle atrophy, and loss of ambulation, historically between 6-12 years of age. With the progressive weakness affecting also the upper body, DMD patients will develop progressive scoliosis, secondary to weakness of the para-spinal muscles, which also affect respiratory function.(Muntoni, 2003) (Eagle et al., 2007)

Despite the major advances in understanding the underlying pathogenic mechanisms of the disease, no cure is currently available for DMD. However, as a result of the implementation of internationally agreed standardised guidelines of clinical care (Bushby et al., 2010a, Bushby et al., 2010b), the mean age of loss of ambulation has shifted in the mid-teens (Bello et al., 2016), and life expectancy for DMD patients has now shifted well into adulthood with reported age of death in the 30s-40s (Ishikawa et al., 2011, Moxley et al., 2010, Eagle et al., 2002).

The standards of care for DMD encompass a multidisciplinary medical, surgical and rehabilitation approach of the multiple symptoms of the disease. The key interventions, which have profoundly altered the disease course, include respiratory support, surgery for scoliosis, (Eagle et al., 2002) physiotherapy and intervention with glucocorticoids. (Manzur et al., 2008, Ricotti et al., 2013). Furthermore, in recent years a number of experimental therapeutic approaches have been developed including compounds aiming at restoring the absent dystrophin protein in muscles, and mostly targeting specific mutations of the *dystrophin* gene, such as exon skipping mediated by antisense oligomer nucleotides (Goemans et al., 2011, Cirak et al., 2011, Voit et al., 2014, Mendell et al., 2013); read-through of intragenic stop codon mutations by small molecules (Welch et al., 2007, Hirawat et al., 2007); other non-mutation dependent therapeutic approaches (Buyse et al., 2013) (Nelson et al., 2014) (Tinsley et al., 2011); and viral-mediated gene therapy, which can function as replacement therapy, for example using a mini-dystrophin (Koo et al., 2011, Odom et al., 2011, Gregorevic et al., 2008).

Clinical trial designs for DMD currently rely on outcome measures of muscle function and strength, which get evaluated during clinical appointments. (Mazzone et al., 2010, McDonald et al., 2010) (Mayhew et al., 2013) Such tests require attendance to hospital appointments and results can be influenced by patient motivation and level of tiredness on the day. Furthermore, these measures are often based on human judgement, that even for expert clinicians is subjective (it varies between practitioners) and varies across repetition within the same clinician. As a consequence, a larger sample size and longer duration of clinical trials are required, often delaying access to new drugs or creating a disincentive for industries to invest in drug development for this condition.

Due to the very nature of the disease, it is undisputable that accurate and precise tracking of movement kinematics would therefore provide an important insight to understand disease

progression and response to therapy. Furthermore, data from natural activities of daily living (ADL), in other words data captured "in-the-wild" would be even more informative and insightful as a reflection of the meaningfulness of treatment on daily activities.

Background and scientific rational

Imperial College London (ICL) has designed a low-cost, non-invasive, compact and wireless system, which can be easily attached to human body parts for the recording of body motion in a natural environment. The sensor unit (i.e. ETHO) consists of electronic components that already exist in the market. In addition, using the wireless connectivity, each sensor can transmit the collected data to a nearby computer for processing and storage and also communicate with the other sensor nodes for providing synchronisation and calibration information. Each sensor node works independently from the others to enable custom- made networks with different numbers of nodes for specialised research experiments.

This sensor-system has been studied on a number of healthy volunteers of adult age and on adult subjects affected by Friedreich's Ataxia, a rare progressive neurodegenerative disease. The study on Friedreich's Ataxia has revealed a number of kinetic biomarkers that correlated with the Scale for the Assessment and Rating of Ataxia, mostly used in clinical settings to evaluate disease progression. (Gavriel, 2015) These biomarkers included: movement interval markers, stillness interval markers and kinematic intensity markers, which were extracted from patient's sleep behavioural patterns. (Gavriel, 2015) In addition, ICL is exploring the use of the Apple Watch as conveniently packaged system for the Inertial Measurement Unit (IMU) data collection. This device has the added benefit that it tracks ambience properties and heart rate information.

Leveraging on such technology, this study allows monitoring patients' movement behaviour in their daily life and in the environment that matters most to them – in their home and around there. Instead of relatively short time measurements in the clinic, wearable technology and motion capture technology will allow to monitor patient movements continuously. Thus, boosting the amount of data, which is used to determine subtle changes in movement ability, the aim is also to develop biomarker which are sensitive, reproducible, able to pick up changes earlier, and that can be measured more frequently.

Two novel measures are going to be collected at two levels of kinematic resolution:

1. The "suit" system – A high-resolution measurement sensor system involving a motion capture suit that tracks and reconstructs full skeletal movement.
The sensors detect rotations (of joints, by using a built-in compass and to keeps track accurately of these over time (thus detect the very weak earth magnetic field). Up to 17 sensors are embedded in wearable straps. The data is live streamed wirelessly to a base station. It is a standard technique that we tested in clinical settings, in-the-home and in outside the lab. In addition, a camera is used next to the sensor so to be able to capture with precision the activities performed and determine the relationship between the data acquired by the sensors to specific activities.

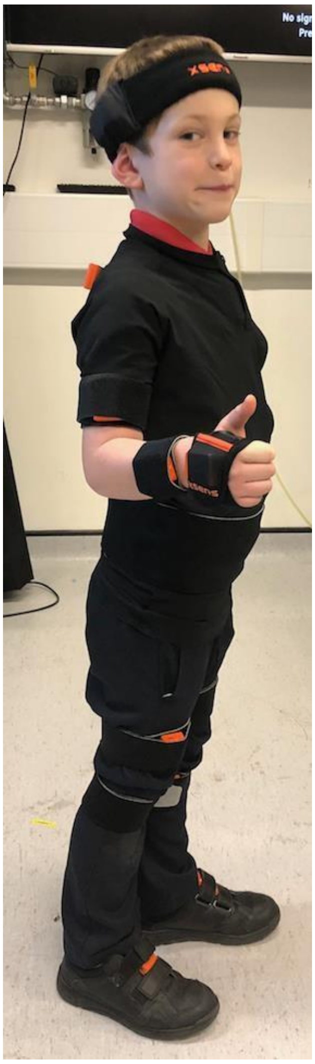

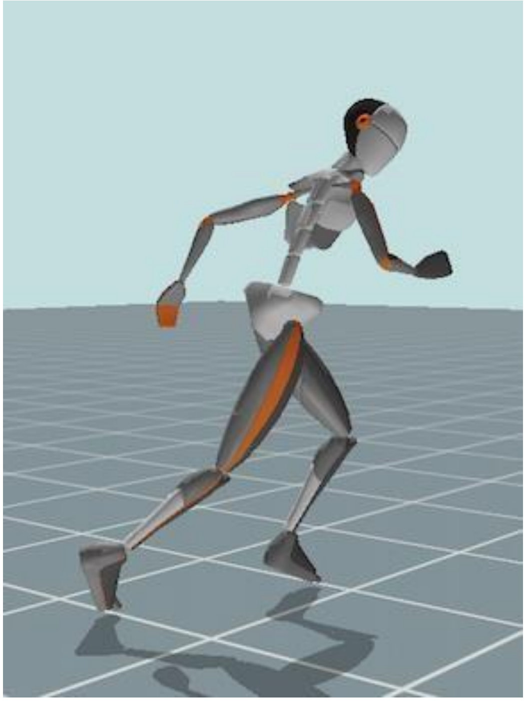

Figure 1 (left): the high resolution "suit" system, example of how the sensors can be placed with elastic straps. (right) Avatar reconstruction of movement.

Quantitatively recording movement data in high resolution, will allow detecting small changes in movement patterns that may be overlooked in a clinical assessment or by human eye. This high resolution behavioural data will be analysed using state-of-the –art machine learning and data science methods that we have developed. Using these pattern recognition and classification algorithms we will be able to extract objective measures of disease progression from patient behaviour data. This will allow deriving new clinical end-points that capture individual variations in disease progression and variation in movement performance throughout the day. Crucially, data will be integrated data from different types of sensors (e.g. movement of the arms and legs, heart rate), to derive a polymarker of disease state.

During a selection of the assessments, specifically the 6MWT, some participants will be asked to wear a fNIRS sensors, placed on one arm and/or leg to measure muscle perfusion during assessment period. fNIRS which will allow for accurate measurement of the blood oxygenation of the muscle group upon which it is placed. They will be placed directly onto the skin with medical tape.

2. Low-resolution “bracelets” will be worn by the boys on a daily basis, and for this purpose Apple Watch devices will be used.
The Apple Watch device has the added benefit that it tracks ambience properties and heart rate information. Key feature is the ability to collect data during a normal school day without the need for additional equipment to be carried by the user aside from the wearable technology.

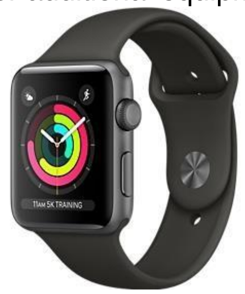

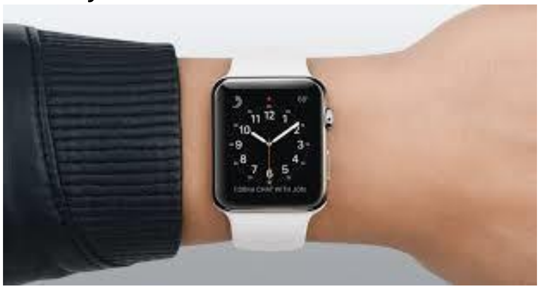

Figure 3: Apple watch device.

Specific aims

The primary objectives of this pilot study are the following:

To investigate kinetic behaviour in subjects affected by DMD with daily recording of body motion in their natural environment (i.e. outdoors and in their home environment, ‘in-the-wild’).
To assess agreement with gold-standard validated functional assessments in clinic.
To build and test a proof-of-concept for next-generation outcome tracking in DMD.

Design and Methodology

Study design

This prospective longitudinal observational study will be conducted in two sites, Great Ormond Street Hospital, and Imperial College London, in collaboration with the Dept. of Computing & Bioengineering, Imperial College London. For clinical amd safety reasons, participants with DMD will be reviewed at GOSH, whereas age mathed healthy controls can have the choice of attending GOSH or ICL.

Enrolment will include up to 30 subjects with a confirmed diagnosis of DMD and up to 30 gender- and age-matched healthy volunteers, who will be enrolled into the study by the clinical PI.

Data from DMD subjects will be collected for 12 months with 3 hospital visits.
Healthy subjects will be evaluated at the beginning and end of the study, with data being collected for one week at each time point.

Inclusion criteria for DMD subjects

- ✦ Written informed assent /consent of patient and parents are obtained, and all are willing and able to comply with the protocol (in the opinion of the Investigator).
- ✦ Clinical diagnosis of DMD that is genetically confirmed by MLPA, full gene sequencing, or any other state of the art diagnostic technique
- ✦ Gender: Male
- ✦ Stable dose of glucocorticoids for 3 months prior to screening and to remain constant throughout the study as per kg basis
- ✦ Internet connectivity: WiFi
- ✦ At least 25 DMD subjects to be ambulant, 6-13 years of age
- ✦ In addition, up to 5 non-ambulant DMD subjects may be enrolled, 12- 18 years of age ✦ Motor function inclusion criteria for ambulant subjects:
  - o 6MWT: 280- 450 meters ○ NSAA total score 20 out of 34 ○ Time rise from supine: 10 seconds
- ✦ Motor function inclusion criteria for non-ambulant subjects:
  - o Unable to walk 10 meter without assistance ○ Score 4 Brooke scale for upper extremity

Exclusion criteria for DMD subjects

- ✦ Cognitive and neuro-behavioural conditions that prevent the subject from performing the actions necessary for the study, in the opinion of the clinical PI
- ✦ Any prior or ongoing history of a clinically significant medical condition (other than DMD) that deemed by the investigator may confound the results of the study.
- ✦ Impaired cardiovascular function (Shortening Fraction <28%, Ejection Fraction <45% on echocardiography)
- ✦ Having a respiratory function predictive of (or requiring) the use of daytime ventilatory support or presenting a Forced Vital Capacity (FVC) <40% of age- and weight-adjusted normal in adolescents)
- ✦ Major surgery within 3 months prior to recruitment or planned orthopaedic surgery for any time during this study which would interfere with the ability to perform outcome measures
- ✦ Participation in any interventional study with the exception of the following: FOR-DMD and the Heart Protection Study after having discussed with PI. Participation in natural history/observational studies is NOT an exclusion criterion.

Recruitment and Informed consent

DMD subjects’ enrolment

Participant recruitment will only commence when the trial has all respective approval letters in place.

Patients and parents will be approached about this study by their respective treating clinician, or by a patient advocacy group. If the patient and his parents are interested, they will be given contact information of the PI/study coordinator so that more information about the study can be supplied. More than two days will be allowed before contacting the patient and parents to follow-

up. Interested patients and parents will then discuss with the PI and the team to review the aims and risks of the study protocol. Patients and parents who are unable to understand the nature of the study will not be allowed to participate.

Healthy subjects' enrolment

Potential subjects will be approached about this study through one or more mass emails to friends and family of employees of UCL and ICL. Each mass email will inform recipients about the dates/times of the study sessions; the purpose and instructions of the study sessions. The study will only enroll age- and gender-matched volunteers as healthy controls.

Informed consent

Consent procedures approved by the Ethics Committee will be followed.

Once a decision to participate has been made, written informed consent will be obtained prior to any study procedures or assessments. If the participant is under 16 years, the signed consent will be given by the parents/guardians, as well as age appropriate assent signed by the potential participants themselves. Participants are considered to be enrolled into the trial following consent.

After consent is obtained, participants will be allocated a unique study number. Eligibility based on the inclusion and exclusion criteria will be confirmed. Any participant who is assessed to be ineligible to continue in the study will be recorded as a screen failure.

Study timeline

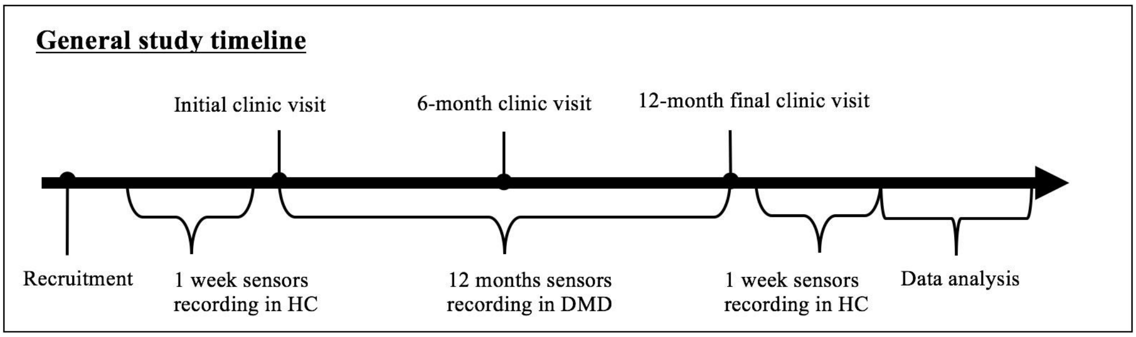

Study procedures

Study procedures for healthy controls

Healthy controls will be attending 2 study visits lasting approximately 5 hours at either Great Ormond Street Hospital or Imperial College London. The high-resolution "suit system" will be tested in a hospital setting and operated in a controlled environment under supervision by members of the clinical research teams. At recruitment and at 12 months HC will be asked to carry out ADL during their stay in hospital (e.g. going up and down the stairs, eating, writing) while

wearing the high resolution 17-node system. The subjects will wear a GoPro a camera to the forehead so to be able to capture with precision the activities performed and determine the relationship between the data acquired by the sensors to specific activities.

At home, the healthy volunteers will wear 4 bracelets (Apple Watch) for a full week once at the beginning of the study and 12 months later. The 4 sensors will be placed in the ankles and wrists and will monitor activities of daily leaving. Only 1 of the sensors will be in direct contact with the skin, allowing continuous heart monitoring. The remaining sensors will be placed in elastic sport bands. Volunteers will be asked to wear the bracelets at least one night during the course of a week.

Volunteers will be given the option to provide a urine samples for biomarker analysis. After providing and additional information sheet and consent form to the families, if the families and the child agree, a total of 30 ml of Urine will be collected at each time point and stored in the UK Biobank according to the SOP included in the appendix.

Families and subjects will be asked to complete web-based User Experience Questionnaire at twice each week (Appendix).

Schedule of Events for healthy controls

Study Assessment	Screening/Baseline hospital visit	Home for a week	12 month hospital visit	Home for a week
Informed consent	x			
Confirm eligibility	x			
Demographic / Medical history/ Height and weight	x		x	
ConMed Assessment	x		x	
Suit system + GoPro[2]	x		x	
4 bracelet-system[3]		x		x
Follow-up call/email[4]		x		x
User experience questionnaire[5]		x		x
Urine collection[6]	X		X	
AE assessment	x	x	x	x

Foot notes to HC schedule of events table

1 Physical examination includes weight and height

2 The 17-sensor system will be set up at the beginning of the hospital visit. The data will be recorded for a maximum of 5 hours whilst in hospital. The child will wear a GoPro on the forehead while performing normal daily activities [3] For continuous monitoring at home day and night

4 Families will be regularly reached out by phone or email

[5] Twice in a week, web-based
[6]Urine collection for biobank (optional)

Study procedures for DMD

DMD participants will be attending three study visits lasting approximately 5 hours at Great Ormond Street Hospital.

During the hospital visits participants will be asked to wear the 17-node wireless ETHO1 sensors system. The system will be tested in a hospital setting and operated in a controlled environment under supervision by members of the clinical research teams.

A screening visit will coincide with a baseline visit provided inclusion criteria are met. At each visit, DMD subjects will undergo all of the following tests and procedures:

- Medical history and medication history
- Height and weight measurement
- Physical examination including vital signs
- Blood test and urine sample for biomarkers
- Measurement of joint end range via goniometry
- Motor functional and strength tests including the following:
    a. Brooke Scale for upper extremity for ambulant and non-ambulant subjects
    b. Performance of Upper Limb scales- for ambulant and non-ambulant subjects
    c. Hand and pinch strength with myotools
    d. North Star Ambulatory Assessment - for ambulant subjects
    e. Time rise from floor from supine -for ambulant subjects
    f. Time to run 10 meters- for ambulant subjects
    g. 6 Minute Walk Test -for ambulant subjects Questionnaire of quality of life

The majority of the above activities are routinely done during their standard clinical appointments.

Measurement of joint end range

Following the NSAA guidelines, range of movement of lower limb (ambulant subjects only) and upper limb will be assessed and measured with goniometry at each motor function evaluation.

Brooke scale for upper extremity

The Brooke scale was designed to assess the upper extremity function. The grades of the Brooke scale range from 1 to 6: grade 1 means that the subject can elevate his arms full range to the head with the arms straight; grade 2 means that the shoulder strength is insufficient to elevate his arms, and the subject needs to flex the elbow to elevate the arms; in grades 3 and 4, the subject is unable to elevate the shoulders but can raise his hands to the mouth with or without weight respectively; grade 5 refers to the subject being unable to raise his hands to the mouth and only some hand movement exists; and grade 6 refers to no useful function of hands. (Brooke et al., 1981)

Performance of the Upper Limb (v2.0)

The Performance of Upper Limb (PUL) was specifically designed for DMD to evaluate the progression of weakness and natural history of functional decline in DMD. Psychometric methods were employed to create a viable scale to enable a clinician-reported outcome assessment tool that can establish clinical meaningfulness and relevance of activities of daily living concerning arm function (Mayhew et al., 2013).

Myoset

The Myoset consists of very sensitive myometers (i.e. MyoPinch and MyoGrip) that evaluate hand grip and finger pinch strength at each hospital visit (Seferian et al., 2015)

North Star Ambulatory Assessment and timed tests

The NorthStar Ambulatory Assessment (NSAA) is a clinician-administered scale that rates subject performance on various functional activities (Mayhew et al., 2011, Mazzone et al., 2010). During this assessment, subjects will be asked to perform 17 different functional activities including: a 10-meter walk/run, rising from a sit to stand, standing on 1 leg, ascending a box step, descending a box step, rising from lying to sitting, rising from the floor, lifting the head, standing on heels, and jumping. Patients will be graded as follows: 2 = normal, no obvious modification of activity; 1 = modified method but achieves goal independent of physical assistance from another; and 0 = unable to achieve goal independently.

6-Minute Walk Test

The 6-minute walk test (6MWT) is an assessment of ambulation using a modified version of the American Thoracic Society (ATS) guidelines. The modified version has been used in numerous clinical trials in DMD. The test requires the child to walk for 6 minutes along a taped 25-meter course. (McDonald et al., 2010, Mazzone et al., 2013).

Patient-reported outcome and quality of life questionnaire

To better understand the clinical meaningfulness of changes, subject and families/caregivers will be asked to complete a self-reported questionnaire The Paediatric Outcomes Data Collection Instrument (PODCI) with a 6-month interval (Lerman et al., 2005) (Appendix)

User Experience Questionnaire

Families and subjects will be asked to complete web-based User Experience Questionnaire at least once each week (Appendix).

Bracelets (Apple Watch) home recording during daily activities

DMD subjects will be asked to wear a reduced set of sensors consisting of 2 bracelets/apple watches (non-ambulant subjects) or 4 bracelets/apple watches (ambulant subjects) during daily activities including and not limited to home and school. The sensors will be strapped to the wrists

of non-ambulant subjects and to the ankles and wrists of ambulant subjects. Only 1 apple watch will be strapped in direct contact of the skin to allow heart rate monitoring. The remaining sensors will be placed in elastic sport bands.

DMD subjects or their caregivers will also be asked to record any special events (e.g. falls) during the recording. At least once a week the sensors will be worn also during sleep. Battery will be recharged via USB plug during the night or when the sensors are not in use. Patient and his family will be trained by study staff regarding tool manipulation, battery charge, troubleshooting in case of technical problems.

The data will be streamed to a remote processing service via a laptop.

Families and subjects will be asked to complete web-based User Experience Questionnaire on a weekly basis (Appendix)

Suit system in clinic recording

At each clinic visit (baseline, 6 and 12 months), DMD subjects will wear the full ETHO system consisting of 17 sensors, which will be strapped as illustrated in figure 1. Subjects will wear the complete sensor-set during their full stay in hospital and while performing functional assessments, which will be videoed, if consent is given. In addition, during the duration of the hospital stay, DMD boys will be asked to move around and perform activities of daily living, such as eating a snack, going up and down the stairs. The subjects will wear a GoPro (a camera) on the forehead so to be able to capture with precision the activities performed and determine the relationship between the data acquired by the sensors to specific activities.

Laboratory assessments for Biobank storage

After providing and additional information sheet and consent form to the families, if the families and the child agree, a total of 10 ml of blood and 30 ml of Urine will be collected at each time point and stored in the UK Biobank according to the SOP included in the appendix.

In addition to the above study procedures, participants with DMD will be given the option of participating in the study in a combined Suit and Apple watch motion sensor data collection only. This would mean that participants followed the above procedure for suit data collection, whilst wearing 4 apple watches but not participate in the daily data collection at home.

Schedule of Events for DMD

**Study Assessment**	**Screening/Baseline hospital visit**	**Home for 6 months**	**6 month hospital visit**	**Home For 6 months**	**12 months hospital visit**
Informed consent	x				
Confirm eligibility	x				

Demographic<br>Medical history	x		x		x
ConMed Assessment	x		x		x
Complete physical examination[1]	x		x		x
Muscle force and function assessment[2]	x		x		x
Blood[3] and Urine collection	x				x
Suit system + fNIR sensors[4]	x		x		x
PODCI Questionnaire		x		x	
Bracelets system[5]		X-->			
Follow-up call/email[6]		x		x	
User experience questionnaire[7]		x		x	
AE assessment	x	x	x	x	x

Foot notes to DMD schedule of events table

Training

The PI and physiotherapists in this study will be qualified and trained to use the sensors. The study team will be regularly in touch with the families to ensure that the sensors are used in the appropriate way.

Data Management

The handling of all data on the CRFs will be the responsibility of the Principal Investigator. It will be the responsibility of the investigator to ensure the accuracy of all data entered in the CRFs.

---

[1] Physical examination includes weight, height and BP

[2] To be performed in the order according to study manual; the assessments will be videos (optional)

[3] Coinciding with clinical bloods, twice in a year (optional)

[4] The 17-sensor system will be set up at the beginning of the hospital visit. The data will be recorded for a maximum of 5 hours whilst in hospital. Some children will also be asked to wear fNIRS sensors at the same time

[5] For continuous monitoring at home (2 for non-ambulant and 4 for ambulant)

[6] Families will be regularly reached out by phone or email

[7] Once a week, web-based

The delegation log will identify all those personnel with responsibilities for data collection and handling, including those who have access to the trial database. Once all the subject data has been collected, the analysis and reporting will be conducted. Details of these subjects will, however, be referenced in the final report.

Any data existing for subjects who have received the investigational device, who withdraw voluntarily or who are withdrawn from the clinical investigation, will be used in the final analysis. The inclusion of partial data will be documented in the final report. The final report will be the responsibility of the investigator.

The investigator institution will permit trial-related monitoring, audits, REC review, and regulatory inspection, providing direct access to source data/documents. Trial participants are informed of this during the informed consent discussion. Participants will consent to provide access to their medical notes.

Data Confidentiality

All data will be handled in accordance with the UK Data Protection Act 1998. The CRFs will not bear the subject's name or other personal identifiable data. The subject's initials, date of birth and Investigation identification number, will be used for identification. Subjects will be assigned an Investigation identification number by the study site sequentially upon enrolment into the study. The study site will maintain a master Subject Identification Log. The in-clinic video recordings will be labelled with the subject's study ID number, and the date and time of the recording. While the recording may include full facial pictures, the subject's name will not be used. The recording will be stored in a locked file cabinet. The code linking the subject's study ID will be kept in a separate secure location. The recordings will be kept indefinitely at GOSH and a copy sent to Imperial College. Other measurements and questionnaires collected at these visits will also be similarly labelled and stored. GDPR will be followed throughout this study.

Record keeping and archiving

Archiving will be authorised by the Sponsor following submission of the end of study report. Chief and Principal Investigators are responsible for the secure archiving of essential Investigation documents as per their Trust policy. All essential documents will be archived for at least 5 years after completion of Investigation. Destruction of essential documents will require authorisation from the Sponsor.

Data Collection and Completion of Case Report Forms

The Principal investigator will be responsible for the timing, accuracy and completeness of a CRF for each individual subject. All entries are to be made in black ink and are to be legible. All corrections made are to be completed by placing a single line through the incorrect data and the individual making the correction must initial and date the correction. Typing correction fluid must not be used. The personal data recorded on all documents will be regarded as confidential.

The Principal investigator must record the subject's participation in this clinical investigation in the subject's hospital notes. In addition, the Principal investigator must keep a separate list of all subjects entered into the clinical investigation showing each subject's name, date of birth and

assigned subject number (for identification purposes). A Subject Identification Log will also be provided in the Investigation Site File to record the subject’s initials and assigned subject number.

All data will be handled in accordance with the UK Data Protection Act 1998. The CRFs will not bear the subject’s name or other personal identifiable data. The subject’s initials, date of birth and trial identification number, will be used for identification.

Retention of Documentation

The Principal investigator will retain all copies of the records for a period of 5 years from the discontinuation of the clinical investigation. In all cases, the Principal investigator must contact the Sponsor prior to disposing of any records related to the clinical investigation. Included in records to be maintained are signed Clinical Investigation Plan, copies of the CRFs, signed consent forms, ethics committee approval letters, product accountability records, correspondence concerning the clinical and any other documents to identify the subjects.

In addition, if the Principal investigator moves/retires, etc., he should provide University College London with the name and address of the person who will look after and be responsible for the clinical investigation related records.

Biostatistics Analysis

The behavioural data collected with both the suit and bracelets will be analysed using stateof-the-art machine learning and data science methods that we developed. Using pattern recognition and classification algorithms we will be able to extract objective measures of disease progression from patient behaviour data. Computer classifiers capable of distinguishing certain types of DMD participants will be developed. This will allow deriving new clinical end-points that capture individual variations in disease progression and variation in movement performance throughout the day. Crucially, data will be integrated data from different types of sensors (e.g. movement of the arms and legs, heart rate), to derive a polymarker of disease state. The aim of this part of the study is to identify and define potentially useful biomarkers, which will involve referencing data from control boys.

In the case of Friedreich's ataxia three main kinetic biomarkers were identified extracted from sleep behaviour and correlated with the Scale for the Assessment and Rating of Ataxia. (Gavriel, 2015)

1. In DMD boys, for each kinetic biomarker we will compare data collected in clinic with that collected in the wild using baseline and 12-month follow-up measures. We will account for the repeated measures structure of the data and use suitable transformation for skewed data; previous work has suggested a log transformation may be appropriate.

2. For all DMD boys we will explore the changes in each of the biomarker values over time (how much data will we have – depends on segmentation – which could be more refined for this aspect). For both clinic and wild data, we will plot individual biomarker trajectories for boys to explore the nature of any relationship with time and will use a multilevel model to describe the relationship. All DMD boys will be older than 7 years of age and hence will have started to deteriorate in terms of general motor functioning, as indicated by standard

clinical measures. Similarly using data collected in the wild, we will explore and describe the relationship for the biomarkers over time for healthy controls, who should remain unchanged over the 12 months' study period.

3. Lastly, we will investigate and describe the relationship between changes in the new biomarkers with changes in standard clinical measures, such as NSAA and 6-minute walk test for DMD boys.

This is a pilot study, with small numbers of enrolled subjects and hence there is no formal power calculation.

All data will be analysed with using the statistics package Stata for all my analysis

General Practitioner Involvement

The GP/general paediatrician of participants in the study will be informed with a letter of notification and an information leaflet specific for the child taking part in the study.

Potential Risks for Participants

There are no major invasive procedures involved in this research protocol. The risk is that an extended physiotherapy assessment could cause increased fatigue for individuals. Fatigue in this patient group is a possibility. To minimise this, breaks will be given, as necessary. It is part of normal practice to offer refreshments prior to and after items. Within the protocols for individual assessments there are procedures for managing fatigue. Assessments are designed to minimise position change so as not to unduly fatigue individuals.

Physiotherapists undertaking physical assessments need to be aware of potential harm to themselves whilst delivering the tests. There are no more risks involved in this assessment than any other routine examination. Guidelines exist for lifting and handling to safeguard patients' and therapists' safety whilst conducting these procedures.

Discomfort from venepuncture will be minimised with application of numbing cream prior to procedure (i.e.: Ametop, EMLA). We will also be using our Clinical Research Facilities at GOSH, where additional support for the children is offered with a play therapist and a wide range of toys.

Risks associated with wearing the sensors (bracelets and/or suit) may include mild discomfort.

Additional psychological support to vulnerable families may also be offered, in the event of these families developing emotional problems, as the disease progresses during the study duration.

Potential Benefits for Participants

Participants may not directly benefit from this study, although it is anticipated that evaluation of the data collected will benefit individuals with DMD in the future by assisting in the design of potential clinical trials, further reducing the necessity of invasive procedures as outcome measures, ultimately reducing the time required to access new therapies. The information

collected in the database will lead to a better understanding of the natural course of DMD, which may help in providing information on the course of the disease to future patient groups.

Participants will not receive any payments or reimbursements specifically for taking part in this study, however they will be able to retain the laptop used during the study.
Travel expenses will be reimbursed as long as valid tickets/receipts are shown on appointment days. Lunch will be reimbursed for the parents and child. Healthy volunteers will be offered a £50 voucher as a gesture of gratitude for their participation.

Safety Monitoring plan

The study does not foresee any safety issues, as the patients will undergo assessments that are similar to those used in routine clinical practice the only constraint being that the tests may cause fatigue.

Adverse events

**Term**	**Definition**
Adverse Event (AE)	Any untoward medical occurrence, unintended disease or injury, or untoward clinical signs (including abnormal laboratory findings) in subjects, users or other persons, whether or not related to the investigational medical device.<br>Note 1: This definition includes events related to the investigational medical device or the comparator<br>Note 2: This definition includes events related to the procedures involved<br>Note 3: For users or other persons, this definition is restricted to events related to investigational medical devices
Serious Adverse Event (SAE)	Any adverse event that:<br>• Led to death,<br>• Led to serious deterioration in the health of the subject, that either resulted in<br>• a life-threatening illness or injury, or<br>• a permanent impairment of a body structure or a body function, or<br>• in-patient or prolonged hospitalisation, or<br>• medical or surgical intervention to prevent life-threatening illness or injury or permanent impairment to a body structure or a body function,

An adverse event does not include:

- Medical or surgical procedures; the condition that leads to the procedure is an adverse event.
- Pre-existing disease, conditions, or laboratory abnormalities present at the start of the study that do not worsen in frequency or intensity.
- Situations where an untoward medical occurrence has not occurred (e.g., hospitalizations for cosmetic or elective surgery or social/convenience admissions);
- The disease being studied or signs/symptoms associated with the disease unless more severe than expected for the subject’s condition.
- Expected post-operative course

Severity

Category	Definition
Mild	The adverse event does not interfere with the subject's daily routine, and does not require intervention; it causes slight discomfort
Moderate	The adverse event interferes with some aspects of the subject's routine, or requires intervention, but is not damaging to health; it causes moderate discomfort
Severe	The adverse event results in alteration, discomfort or disability which is clearly damaging to health<br>Note: A severity rating of severe does not necessarily categorise the event as an SAE.

Causality

The assessment of relationship of adverse events to the study procedure and the investigational device will be a clinical decision based on all available information at the time of the completion of the case report form. The following categories will be used to define the causality of the adverse event:

Category	Definition
Yes	There is evidence to suggest a causal relationship, and the influence of other factors is unlikely
Possibly	There is some evidence to suggest a causal relationship (e.g. the event occurred within a reasonable time after procedure). However, the influence of other factors may have contributed to the event (e.g. the patient's clinical condition, other concomitant events).
No	There is no evidence of any causal relationship.

Expectedness

Category	Definition
Expected	An adverse event that is consistent with the information about the device listed in this CIP.
Unexpected	An adverse event that is not consistent with the information about the device listed in this CIP.

Reporting of all Adverse Device Effects

Investigator responsibilities shall be as per section d). The sponsor shall keep detailed records of all adverse events relating to the clinical Investigation, which are reported to them by the Investigation investigators. The sponsor shall ensure that all relevant information about a reportable event, which occurs during the course of this clinical Investigation in the United Kingdom, is reported as soon as possible to the relevant ethics committees per their reporting requirements and according to the timelines in section d. Any additional relevant information should be sent within the same time frame as the initial report. The PI is responsible for informing the appropriate ethics committees and other investigators of any reportable events that have occurred with the study device in any clinical investigation according to the guidelines set forth by REC in the country where the clinical investigation is taking place.

Duration of exclusion

No exclusion period is scheduled after enrolment of patients. The study does not include a prolonged follow-up period and no residual effects are expected.

Patients can participate simultaneously in other clinical studies of natural history or description of the disease (no treatment), with the exception of the Heart Protection Study and FOR-DMD.

Management of study drop-outs

Subjects who are unable to perform the tests under the conditions described in the protocol or who are unwilling to complete the study for any reason may be replaced by another patient of a similar age in the initial 6- 8 months of the study.

Definition of End of Trial

The end of trial is the date of the last telephone follow up of the last participant.

Suspension or premature termination of the clinical investigation

Inclusion in the study will be discontinued in any participant who does not wish to continue. In addition, if the device or study procedures are causing undue pain or discomfort in the opinion of the participant or investigator then the subject’s participant will be discontinued. If the device malfunctions or breaks recruitment into the study will be temporarily halted. If the device can be repaired in a short interval then the participants can be rescheduled. If the device is unable to be restored to working order or a new device provided, then the study will need to be discontinued.

All data collected up to this point will still be analysed.

Early Termination of the Clinical Investigation

Both the Sponsor and the Principal investigator reserve the right to terminate the clinical investigation at any time. Should this be necessary, the procedures will be arranged on an individual basis after review and consultation by both parties. In terminating the clinical investigation, the JRO at University College London and the Principal investigator will assure that adequate consideration is given to the protection of the subject’s interests.

Deviations from clinical investigation plan

A deviation is considered a departure from the conditions and principles of GCP in connection with that Investigation; or the CIP relating to that Investigation, as amended from time to time.

The Investigator shall not deviate from this CIP except in situations that affect the subject’s rights, safety and well-being, or the scientific integrity of the clinical investigation.

Procedures for recording, reporting and analysing CIP deviations

If possible, prior approval from the sponsor and REC, if appropriate, shall be obtained by the investigator. All spontaneous CIP deviations shall be recorded and reported to the sponsor as agreed. A deviation log shall be maintained by the study site. Deviations shall be reported to the REC and the Sponsor. All deviations will be included, as required in the final study report.

Ethical Issues

The main ethical issues for this project include the need to attend additional clinic appointments beyond 'routine' and the need to spend minimal time for manipulating the sensors. It is not anticipated that the hospital stays will be longer than 5 hours at each visit, and that strapping the sensors, uploading and transferring of the data will take no longer than 10-15 minutes a day. The patient information sheet will outline all the assessments performed during the duration to the study, and the signed consent form will confirm that the participant has read and understood the information sheet, and so has agreed voluntarily to take part in the study.

No other issues have been identified to arise from this study. Informed consent will be documented using the information sheet and consent form, and the Data Protection Act 1998 will be adhered to as per routine clinical care. All data held and disseminated for research purposes will be anonymised, containing no personal identifiers. The commencement of the study at each UK site will be subject to NHS R&D management approval.

Indemnity

Imperial College London holds negligent harm and non-negligent harm insurance policies which apply to this study/ Imperial College Healthcare NHS Trust holds standard NHS Hospital Indemnity and insurance cover with NHS Litigation Authority for NHS Trusts in England, which apply to this study (delete as applicable)

Sponsor

Imperial College London/Imperial College Healthcare NHS Trust (delete as applicable) will act as the main Sponsor for this study. Delegated responsibilities will be assigned to the NHS trusts taking part in this study.

Dissemination of Results

The results of the study will be published in peer-reviewed scientific journal(s), presented at relevant national and international meetings and reported as part of submissions to regulatory bodies (NHS R&D offices and Research Ethics Committee). All proposed publications will be discussed with Sponsor prior to publishing other than those presented at scientific forums/meetings. Please refer to UCL publication policy.

REFERENCES

BELLO, L., MORGENROTH, L. P., GORDISH-DRESSMAN, H., HOFFMAN, E. P., MCDONALD, C. M., CIRAK, S. & INVESTIGATORS, C. 2016. DMD genotypes and loss of ambulation in the CINRG Duchenne Natural History Study. *Neurology,* 87**,** 401-9.

BROOKE, M. H., GRIGGS, R. C., MENDELL, J. R., FENICHEL, G. M., SHUMATE, J. B. & PELLEGRINO, R. J. 1981. Clinical trial in Duchenne dystrophy. I. The design of the protocol. *Muscle & nerve,* 4**,** 186-97.

BUSHBY, K., FINKEL, R., BIRNKRANT, D. J., CASE, L. E., CLEMENS, P. R., CRIPE, L., KAUL, A., KINNETT, K., MCDONALD, C., PANDYA, S., POYSKY, J., SHAPIRO, F., TOMEZSKO, J. & CONSTANTIN, C. 2010a. Diagnosis and management of Duchenne muscular dystrophy, part 1: diagnosis, and pharmacological and psychosocial management. *Lancet neurology,* 9**,** 77-93.

BUSHBY, K., FINKEL, R., BIRNKRANT, D. J., CASE, L. E., CLEMENS, P. R., CRIPE, L., KAUL, A., KINNETT, K., MCDONALD, C., PANDYA, S., POYSKY, J., SHAPIRO, F., TOMEZSKO, J. & CONSTANTIN, C. 2010b. Diagnosis and management of Duchenne muscular dystrophy, part 2: implementation of multidisciplinary care. *Lancet neurology,* 9**,** 177-89.

BUSHBY, K. M., HILL, A. & STEELE, J. G. 1999. Failure of early diagnosis in symptomatic Duchenne muscular dystrophy. *Lancet,* 353**,** 557-8.

BUYSE, G. M., GOEMANS, N., VAN DEN HAUWE, M. & MEIER, T. 2013. Effects of glucocorticoids and idebenone on respiratory function in patients with duchenne muscular dystrophy. *Pediatric pulmonology,* 48**,** 912-20.

CIRAK, S., ARECHAVALA-GOMEZA, V., GUGLIERI, M., FENG, L., TORELLI, S., ANTHONY, K., ABBS, S., GARRALDA, M. E., BOURKE, J., WELLS, D. J., DICKSON, G., WOOD, M. J., WILTON, S. D., STRAUB, V., KOLE, R., SHREWSBURY, S. B., SEWRY, C., MORGAN, J. E., BUSHBY, K. & MUNTONI, F. 2011. Exon skipping and dystrophin restoration in patients with Duchenne muscular dystrophy after systemic phosphorodiamidate morpholino oligomer treatment: an open-label, phase 2, dose-escalation study. *Lancet,* 378**,** 595-605.

EAGLE, M., BAUDOUIN, S. V., CHANDLER, C., GIDDINGS, D. R., BULLOCK, R. & BUSHBY, K. 2002. Survival in Duchenne muscular dystrophy: improvements in life expectancy since 1967 and the impact of home nocturnal ventilation. *Neuromuscular disorders : NMD,* 12**,** 926-9.

EAGLE, M., BOURKE, J., BULLOCK, R., GIBSON, M., MEHTA, J., GIDDINGS, D., STRAUB, V. & BUSHBY, K. 2007. Managing Duchenne muscular dystrophy--the additive effect of spinal surgery and home nocturnal ventilation in improving survival. *Neuromuscular disorders : NMD,* 17**,** 470-5.

ELLIS, J. A., VROOM, E. & MUNTONI, F. 2013. 195th ENMC International Workshop: Newborn screening for Duchenne muscular dystrophy 14-16th December, 2012, Naarden, The Netherlands. *Neuromuscular disorders : NMD,* 23**,** 682-9.

EMERY, A. E. 2002. The muscular dystrophies. *Lancet,* 359**,** 687-95.

GAVRIEL, C. A. A. C. T., PEDRO RENTE LOURENC¸O, SATHIJI NAGESHWARAN, STAVROS ATHANASOPOULOS, ANASTASIA SYLAIDI, RICHARD FESTENSTEIN, A. ALDO FAISAL.

2015. Kinematic body sensor networks and behaviourmetrics for objective efficacy measurements in neurodegenerative disease drug trials. *Wearable and Implantable Body Sensor Networks (BSN), 2015 IEEE 12th International Conference on*.

GOEMANS, N. M., TULINIUS, M., VAN DEN AKKER, J. T., BURM, B. E., EKHART, P. F., HEUVELMANS, N., HOLLING, T., JANSON, A. A., PLATENBURG, G. J., SIPKENS, J. A., SITSEN, J. M., AARTSMA-RUS, A., VAN OMMEN, G. J., BUYSE, G., DARIN, N., VERSCHUUREN, J. J., CAMPION, G. V., DE KIMPE, S. J. & VAN DEUTEKOM, J. C. 2011. Systemic administration of PRO051 in Duchenne's muscular dystrophy. *The New England journal of medicine,* 364**,** 1513-22.

GREGOREVIC, P., BLANKINSHIP, M. J., ALLEN, J. M. & CHAMBERLAIN, J. S. 2008. Systemic microdystrophin gene delivery improves skeletal muscle structure and function in old dystrophic mdx mice. *Molecular therapy : the journal of the American Society of Gene Therapy,* 16**,** 657-64.

HATHOUT, Y., CONKLIN, L. S., SEOL, H., GORDISH-DRESSMAN, H., BROWN, K. J., MORGENROTH, L. P., NAGARAJU, K., HEIER, C. R., DAMSKER, J. M., VAN DEN ANKER, J. N., HENRICSON, E., CLEMENS, P. R., MAH, J. K., MCDONALD, C. & HOFFMAN, E. P. 2016. Serum pharmacodynamic biomarkers for chronic corticosteroid treatment of children. *Sci Rep,* 6**,** 31727.

HIRAWAT, S., WELCH, E. M., ELFRING, G. L., NORTHCUTT, V. J., PAUSHKIN, S., HWANG, S., LEONARD, E. M., ALMSTEAD, N. G., JU, W., PELTZ, S. W. & MILLER, L. L. 2007. Safety, tolerability, and pharmacokinetics of PTC124, a nonaminoglycoside nonsense mutation suppressor, following single- and multiple-dose administration to healthy male and female adult volunteers. *Journal of clinical pharmacology,* 47**,** 430-44.

ISHIKAWA, Y., MIURA, T., AOYAGI, T., OGATA, H., HAMADA, S. & MINAMI, R. 2011. Duchenne muscular dystrophy: survival by cardio-respiratory interventions. *Neuromuscular disorders : NMD,* 21**,** 47-51.

KOO, T., OKADA, T., ATHANASOPOULOS, T., FOSTER, H., TAKEDA, S. & DICKSON, G. 2011. Long-term functional adeno-associated virus-microdystrophin expression in the dystrophic CXMDj dog. *The journal of gene medicine,* 13**,** 497-506.

LERMAN, J. A., SULLIVAN, E., BARNES, D. A. & HAYNES, R. J. 2005. The Pediatric Outcomes Data Collection Instrument (PODCI) and functional assessment of patients with unilateral upper extremity deficiencies. *J Pediatr Orthop,* 25**,** 405-7.

MANZUR, A. Y., KINALI, M. & MUNTONI, F. 2008. Update on the management of Duchenne muscular dystrophy. *Archives of disease in childhood,* 93**,** 986-90.

MAYHEW, A., CANO, S., SCOTT, E., EAGLE, M., BUSHBY, K. & MUNTONI, F. 2011. Moving towards meaningful measurement: Rasch analysis of the North Star Ambulatory Assessment in Duchenne muscular dystrophy. *Developmental medicine and child neurology,* 53**,** 535-42.

MAYHEW, A., MAZZONE, E. S., EAGLE, M., DUONG, T., ASH, M., DECOSTRE, V., VANDENHAUWE, M., KLINGELS, K., FLORENCE, J., MAIN, M., BIANCO, F., HENRIKSON, E., SERVAIS, L., CAMPION, G., VROOM, E., RICOTTI, V., GOEMANS, N., MCDONALD, C. & MERCURI, E. 2013. Development of the Performance of the Upper

Limb module for Duchenne muscular dystrophy. *Developmental medicine and child neurology,* 55**,**
1038-45.

MAZZONE, E., MARTINELLI, D., BERARDINELLI, A., MESSINA, S., D'AMICO, A., VASCO, G., MAIN, M., DOGLIO, L., POLITANO, L., CAVALLARO, F., FROSINI, S., BELLO, L., CARLESI,
A., BONETTI, A. M., ZUCCHINI, E., DE SANCTIS, R., SCUTIFERO, M., BIANCO, F., ROSSI,
F., MOTTA, M. C., SACCO, A., DONATI, M. A., MONGINI, T., PINI, A., BATTINI, R., PEGORARO, E., PANE, M., PASQUINI, E., BRUNO, C., VITA, G., DE WAURE, C., BERTINI, E. & MERCURI, E. 2010. North Star Ambulatory Assessment, 6-minute walk test and timed items in ambulant boys with Duchenne muscular dystrophy. *Neuromuscular disorders : NMD,* 20**,** 712-6.

MAZZONE, E. S., PANE, M., SORMANI, M. P., SCALISE, R., BERARDINELLI, A., MESSINA, S., TORRENTE, Y., D'AMICO, A., DOGLIO, L., VIGGIANO, E., D'AMBROSIO, P., CAVALLARO,
F., FROSINI, S., BELLO, L., BONFIGLIO, S., DE SANCTIS, R., ROLLE, E., BIANCO, F., MAGRI, F., ROSSI, F., VASCO, G., VITA, G., MOTTA, M. C., DONATI, M. A., SACCHINI, M., MONGINI, T., PINI, A., BATTINI, R., PEGORARO, E., PREVITALI, S., NAPOLITANO, S., BRUNO, C., POLITANO, L., COMI, G. P., BERTINI, E. & MERCURI, E. 2013. 24 month longitudinal data in ambulant boys with Duchenne muscular dystrophy. *PloS one,* 8**,** e52512.

MCDONALD, C. M., HENRICSON, E. K., HAN, J. J., ABRESCH, R. T., NICORICI, A., ELFRING, G.
L., ATKINSON, L., REHA, A., HIRAWAT, S. & MILLER, L. L. 2010. The 6-minute walk test as a new outcome measure in Duchenne muscular dystrophy. *Muscle & nerve,* 41**,** 500-10.

MENDELL, J. R., RODINO-KLAPAC, L. R., SAHENK, Z., ROUSH, K., BIRD, L., LOWES, L. P., ALFANO, L., GOMEZ, A. M., LEWIS, S., KOTA, J., MALIK, V., SHONTZ, K., WALKER, C. M., FLANIGAN, K. M., CORRIDORE, M., KEAN, J. R., ALLEN, H. D., SHILLING, C., MELIA, K. R., SAZANI, P., SAOUD, J. B. & KAYE, E. M. 2013. Eteplirsen for the treatment of Duchenne muscular dystrophy. *Annals of neurology,* 74**,** 637-47.

MOXLEY, R. T., 3RD, PANDYA, S., CIAFALONI, E., FOX, D. J. & CAMPBELL, K. 2010. Change in natural history of Duchenne muscular dystrophy with long-term corticosteroid treatment: implications for management. *Journal of child neurology,* 25**,** 1116-29.

MUNTONI, A. E. A. F. 2003. *Duchenne Muscular Dystrophy*, Oxford University Press.

NELSON, M. D., RADER, F., TANG, X., TAVYEV, J., NELSON, S. F., MICELI, M. C., ELASHOFF, R. M., SWEENEY, H. L. & VICTOR, R. G. 2014. PDE5 inhibition alleviates functional muscle ischemia in boys with Duchenne muscular dystrophy. *Neurology,* 82**,** 208591.

ODOM, G. L., GREGOREVIC, P., ALLEN, J. M. & CHAMBERLAIN, J. S. 2011. Gene therapy of mdx mice with large truncated dystrophins generated by recombination using rAAV6. *Molecular therapy : the journal of the American Society of Gene Therapy,* 19**,** 36-45.

RICOTTI, V., RIDOUT, D. A., SCOTT, E., QUINLIVAN, R., ROBB, S. A., MANZUR, A. Y. & MUNTONI, F. 2013. Long-term benefits and adverse effects of intermittent versus daily glucocorticoids in boys with Duchenne muscular dystrophy. *Journal of neurology, neurosurgery, and psychiatry,* 84**,** 698-705.

SEFERIAN, A. M., MORAUX, A., ANNOUSSAMY, M., CANAL, A., DECOSTRE, V., DIEBATE, O., LE MOING, A. G., GIDARO, T., DECONINCK, N., VAN PARYS, F., VEREECKE, W., WITTEVRONGEL, S., MAYER, M., MAINCENT, K., DESGUERRE, I., THEMAR-NOEL, C.,
CUISSET, J. M., TIFFREAU, V., DENIS, S., JOUSTEN, V., QUIJANO-ROY, S., VOIT, T.,

HOGREL, J. Y. & SERVAIS, L. 2015. Upper limb strength and function changes during a one-year follow-up in non-ambulant patients with Duchenne Muscular Dystrophy: an observational multicenter trial. *PloS one,* 10**,** e0113999.

THEADOM, A., RODRIGUES, M., ROXBURGH, R., BALALLA, S., HIGGINS, C., BHATTACHARJEE, R., JONES, K., KRISHNAMURTHI, R. & FEIGIN, V. 2014. Prevalence of muscular dystrophies: a systematic literature review. *Neuroepidemiology,* 43**,** 259-68.

TINSLEY, J. M., FAIRCLOUGH, R. J., STORER, R., WILKES, F. J., POTTER, A. C., SQUIRE, S. E., POWELL, D. S., COZZOLI, A., CAPOGROSSO, R. F., LAMBERT, A., WILSON, F. X., WREN, S. P., DE LUCA, A. & DAVIES, K. E. 2011. Daily treatment with SMTC1100, a novel small molecule utrophin upregulator, dramatically reduces the dystrophic symptoms in the mdx mouse. *PloS one,* 6**,** e19189.

VOIT, T., TOPALOGLU, H., STRAUB, V., MUNTONI, F., DECONINCK, N., CAMPION, G., DE KIMPE, S. J., EAGLE, M., GUGLIERI, M., HOOD, S., LIEFAARD, L., LOURBAKOS, A., MORGAN, A., NAKIELNY, J., QUARCOO, N., RICOTTI, V., ROLFE, K., SERVAIS, L., WARDELL, C., WILSON, R., WRIGHT, P. & KRAUS, J. E. 2014. Safety and efficacy of drisapersen for the treatment of Duchenne muscular dystrophy (DEMAND II): an exploratory, randomised, placebo-controlled phase 2 study. *The Lancet. Neurology,* 13**,** 987-96.

WELCH, E. M., BARTON, E. R., ZHUO, J., TOMIZAWA, Y., FRIESEN, W. J., TRIFILLIS, P., PAUSHKIN, S., PATEL, M., TROTTA, C. R., HWANG, S., WILDE, R. G., KARP, G., TAKASUGI, J., CHEN, G., JONES, S., REN, H., MOON, Y. C., CORSON, D., TURPOFF, A.
A., CAMPBELL, J. A., CONN, M. M., KHAN, A., ALMSTEAD, N. G., HEDRICK, J., MOLLIN, A., RISHER, N., WEETALL, M., YEH, S., BRANSTROM, A. A., COLACINO, J. M., BABIAK, J., JU, W. D., HIRAWAT, S., NORTHCUTT, V. J., MILLER, L. L., SPATRICK, P., HE, F., KAWANA, M., FENG, H., JACOBSON, A., PELTZ, S. W. & SWEENEY, H. L. 2007. PTC124 targets genetic disorders caused by nonsense mutations. *Nature,* 447**,** 87-91.

APPENDIX

USER EXPERIENCE QUESTIONNAIRE

To be completed by the subject or his parent/guardian Please circle the correct answer.

1. How old are you/ is your son?
   6-8,   9-12,   13-15,   16-18

2. What best describes your motor function / that of your son?
   Ambulant
   Ambulant but requiring wheelchair at times
   Ambulant only indoors
   Non-ambulant

Given your experience …

3. Would you wear a device that is visible to others?   Yes/Maybe/No/Don't know

4. Would you wear a device that is concealed in your clothing?   Yes/Maybe/No/Don't know

5. Would you wear an implanted device?   Yes/Maybe/No/Don't know

6. Would you allow continuous monitoring 24 hrs a day?   Yes/Maybe/No/Don't know

7. How long would you be willing to wear such a device over a 24 hr interval again? Up to 1 day /Up to 1 week/Up to 1 month/Up to 6 months /More than 6 months

8. How long should the device be able to work before the battery needs to be charged again? Up to 1 day /Up to 1 week/Up to 1 month/Up to 6 months /More than 6 months

9. Would you allow data to be sent away and analysed or stored on a database? Yes/Maybe/No/Don't know

10. Would you consider wearing a device to monitor how active you are?
    Yes/Maybe/No/Don't know

11. Would you use a device that you had to apply yourself without assistance? Yes/Maybe/No/Don't know

12. Would you like the device to determine the level of your condition (e.g., mild or severe)?
    Yes/Maybe/No/Don't know

13. How long should it take to put the sensor device on?

Less than 1 min/Less than 5 min/Less than 10 min/Less than 20 min/Less than 30 min/more than 30 min

14. Would you wear a device that would tell the clinician if you did not wear it often enough?
Yes/Maybe/No/Don't know

15. Would you spend time learning how to use a new wearable device? Yes/Maybe/No/Don't know

16. Would you wear an implant that has a sensor incorporated in it? Yes/Maybe/No/Don't know

17. Please rate the importance of each of the the following statements on a scale of 1 to 10, with 10 being the highest.

A body worn device should...

...be comfortable
...be compact (light and small)
...be discreet
...be easy to attach to the body
...be recyclable
...be reliable
...be simple to operate (and maintain)
...blend in with different types of clothing to be worn with the device
...give instant feedback
...have clear and readable instructions
...increase the accuracy of current clinical procedures
...minimise incorrect use of the system
...motivate the people using it
...not affect normal daily behaviour
...not detach from patient unless needed
...provide clear and useful results
...provide positive feedback to patients
...reduce travel to clinics and hospitals
...speed up currently used clinical procedure

PODCI

Pediatric

Outcomes Questionnaire

Developed by:

American Academy of Orthopaedic Surgeons®

Pediatric Orthopaedic Society of North America

American Academy of Pediatrics

Shriner's Hospitals

To be completed by the parent for children 2 – 10 years old

Based on the Version 2.0 Pediatrics-Parent/Child Outcomes Intrument

Also commonly referred to as the PODCI ("Pediatric Outcomes Data Collection Instrument")

Revised, renumbered, reformatted August 2005

Adolescent (self reported)

Outcomes Questionnaire

Developed by:
American Academy of Orthopaedic Surgeons®
Pediatric Orthopaedic Society of North America
American Academy of Pediatrics
Shriner's Hospitals

To be completed by adolescents 11 – 18 years old.

Based on the Version 2.0 Pediatrics–Parent/ Adolescent Outcomes Instrument
Also commonly referred to as the PODCI ("Pediatric Outcomes Data Collection Instrument")
Revised, renumbered, reformatted August 2005

SOP for Plasma/Urine collection for Biobank

Please also notify UCL Biobank technician (Pierpaolo Ala) by e-mail: ich.cnmdbiobank@ucl.ac.uk

For plasma samples, blood (total volume of 10 ml at each time-point) will be collected in 3 x 5ml plastic lavender-top vacutainer tubes containing spray-dried K2 EDTA, without any liquid additives, mixed 5 times by inversion, and placed on ice. Samples will be centrifuged in a 4ºC pre-cooled centrifuge at 2000 x g for 15 minutes. Plasma will be collected and aliquoted as 1ml in cryogenic tubes affixed with cryogenic labels (up to 8 x 1ml aliquots). All samples should be stored at -80°C.

For urine samples, collect 15-30ml whole urine in 10-mL urine monovette (Sarstedt, Nuembrecht, Germany) containing 3.54 g of guanidinium thiocyanate (GTC).
Send immediately to UCL Biobank technician (Pierpaolo Ala) at room temperature to arrive no later than 24 hours after withdrawal.
