# Supplementary material for: Wearable full-body motion tracking of activities of daily living predicts disease trajectory in Duchenne muscular dystrophy
Source: Nat Med. 2023 Jan 19;29(1):95–103. doi: 10.1038/s41591-022-02045-1 (PMC9873561; doi:10.1038/s41591-022-02045-1)
Supplement: Supplementary file 2 — Reporting Summary [file 41591_2022_2045_MOESM2_ESM.pdf]

nature portfolio

Corresponding author(s): A.Aldo Faisal

Last updated by author(s): 06/09/2022

Reporting Summary

Nature Portfolio wishes to improve the reproducibility of the work that we publish. This form provides structure for consistency and transparency in reporting. For further information on Nature Portfolio policies, see our Editorial Policies and the Editorial Policy Checklist.

Statistics

For all statistical analyses, confirm that the following items are present in the figure legend, table legend, main text, or Methods section.

n/a	Confirmed	
☐	☒	The exact sample size (*n*) for each experimental group/condition, given as a discrete number and unit of measurement
☐	☒	A statement on whether measurements were taken from distinct samples or whether the same sample was measured repeatedly
☐	☒	The statistical test(s) used AND whether they are one- or two-sided<br>*Only common tests should be described solely by name; describe more complex techniques in the Methods section.*
☐	☒	A description of all covariates tested
☐	☒	A description of any assumptions or corrections, such as tests of normality and adjustment for multiple comparisons
☐	☒	A full description of the statistical parameters including central tendency (e.g. means) or other basic estimates (e.g. regression coefficient) AND variation (e.g. standard deviation) or associated estimates of uncertainty (e.g. confidence intervals)
☐	☒	For null hypothesis testing, the test statistic (e.g. *F*, *t*, *r*) with confidence intervals, effect sizes, degrees of freedom and *P* value noted<br>*Give P values as exact values whenever suitable.*
☒	☐	For Bayesian analysis, information on the choice of priors and Markov chain Monte Carlo settings
☒	☐	For hierarchical and complex designs, identification of the appropriate level for tests and full reporting of outcomes
☒	☐	Estimates of effect sizes (e.g. Cohen's *d*, Pearson's *r*), indicating how they were calculated

Our web collection on statistics for biologists contains articles on many of the points above.

Software and code

Policy information about availability of computer code

Data collection	Xsens MVN Analyze 2018, Xsens MVN Analyze 2019
Data analysis	MATLAB (R2019b). The machine learning code that supports the findings of this study is available at https://doi.org/10.6084/m9.figshare.20102678

For manuscripts utilizing custom algorithms or software that are central to the research but not yet described in published literature, software must be made available to editors and reviewers. We strongly encourage code deposition in a community repository (e.g. GitHub). See the Nature Portfolio guidelines for submitting code & software for further information.

Data

Policy information about availability of data

All manuscripts must include a data availability statement. This statement should provide the following information, where applicable:

- Accession codes, unique identifiers, or web links for publicly available datasets
- A description of any restrictions on data availability
- For clinical datasets or third party data, please ensure that the statement adheres to our policy

The data used in the study are not publicly available due to them containing information that could compromise research participant privacy/consent. Anonymized data can be made available for academic purposes upon request to the corresponding author.

Human research participants

Policy information about studies involving human research participants and Sex and Gender in Research.

Reporting on sex and gender	All participants in the study are males as DMD primarily affects males.
Population characteristics	Baseline variables presented in supplementary table 1
Recruitment	The KineDMD study was advertised in the appropriate National DMD Networks and charities. A member of the KineDMD research team (VS) then approached each family, who had expressed interest in taking part, to assess their suitability to participate. Potential selection biases included patients not meeting the criteria to be recruited into interventional clinical trials (mostly driven by genotype) and younger DMD patients who may be more motivated than older and non-ambulant patients. However, we believe our analysis was not impacted as a wider cohort will only strengthen the results.
Ethics oversight	The present study was conducted with approval from appropriate research ethics committees from host institutions (REC Reference: 18/SW/0012 South West - Cornwall & Plymouth Research Ethics Committee) .

Note that full information on the approval of the study protocol must also be provided in the manuscript.

Field-specific reporting

Please select the one below that is the best fit for your research. If you are not sure, read the appropriate sections before making your selection.

☒ Life sciences ☐ Behavioural & social sciences ☐ Ecological, evolutionary & environmental sciences

For a reference copy of the document with all sections, see nature.com/documents/nr-reporting-summary-flat.pdf

Life sciences study design

All studies must disclose on these points even when the disclosure is negative.

Sample size	Sample size estimates in machine learning are non-trivial as the effect of non-linear operations introduced by the AI systems do not lend themselves to simple statistical power calculations. It is difficult to predict the sample size needed. We have used different ways of estimating these based on best-guess and best-available data. We based our sample size on previous AI work for wearable sensor diagnostics [Gavriel, Constantinos et A. Aldo Faisal. "Kinematic body sensor networks and behaviour metrics for objective efficacy measurements in neurodegenerative disease drug trials." In 2015 IEEE 12th International Conference on Wearable and Implantable Body Sensor Networks (BSN), pp. 1-6. IEEE, 2015.] and more than doubled the size to obtain a sufficient safety factor.
Data exclusions	Data from one subject's visit couldn't be included for analysis as his data files were corrupted. No other subjects were affected by this issue. Part of the data from 4 subjects couldn't be included for reasons detailed in the methods section.
Replication	A stricter leave one subject out cross-validation policy (instead of a leave-one-visit-out) was used to ensure the generalization of the results.
Randomization	As this was a natural history study and not an interventional trial, randomization is not applicable. It is in the nature of the wearables study to collect behaviour from subjects who were aware that they wore sensors.
Blinding	As this was a natural history study and not an interventional trial, blinding is not applicable. It is in the nature of the wearables study to collect behaviour from subjects who were aware that they wore sensors.

Reporting for specific materials, systems and methods

We require information from authors about some types of materials, experimental systems and methods used in many studies. Here, indicate whether each material, system or method listed is relevant to your study. If you are not sure if a list item applies to your research, read the appropriate section before selecting a response.

Materials & experimental systems

n/a	Involved in the study
☒	☐ Antibodies
☒	☐ Eukaryotic cell lines
☒	☐ Palaeontology and archaeology
☒	☐ Animals and other organisms
☒	☐ Clinical data
☒	☐ Dual use research of concern

Methods

n/a	Involved in the study
☒	☐ ChIP-seq
☒	☐ Flow cytometry
☒	☐ MRI-based neuroimaging
